# Vaccination Against RhoC in Prostate Cancer Patients Induces Potent and Long-Lasting CD4^+^ T Cell Responses with Cytolytic Potential in the Absence of Clinical Efficacy: A Randomized Phase II Trial

**DOI:** 10.3390/vaccines13040390

**Published:** 2025-04-05

**Authors:** Sara Fresnillo Saló, Juliane Schuhmacher, Anne Rahbech, Sara Ram Pedersen, Tina Seremet, Valero Andreu Matillas, Anna Schöllhorn, Andreas Røder, Steffen Wad Jørgensen, Klaus Brasso, Cécile Gouttefangeas, Per thor Straten, on behalf of the RhoVac-002 Study Group

**Affiliations:** 1 National Center for Cancer Immune Therapy, Department of Oncology, University Hospital Herlev, 2730 Copenhagen, Denmark; sara.fresnillo.salo@regionh.dk (S.F.S.); anne.rahbech@sund.ku.dk (A.R.); saramped@gmail.com (S.R.P.); tina.juanita.seremet@regionh.dk (T.S.); 2 Institute for Immunology, University of Tübingen, 72076 Tübingen, Germanyandreu-matillas.valero@uni-tuebingen.de (V.A.M.);; 3 Cluster of Excellence iFIT (EXC2180) “Image-Guided and Functionally Instructed Tumor Therapies”, 72076 Tübingen, Germany; 4 Department of Urology, Copenhagen Prostate Cancer Center, Rigshospitalet, University of Copenhagen, 2100 Copenhagen, Denmark; andreas.roeder@regionh.dk (A.R.); klaus.brasso@regionh.dk (K.B.); 5 Rhovac ApS, Agern Allé 3, 2970 Hørsholm, Denmark; swj@rhovac.com; 6 German Cancer Consortium (DKTK) and German Cancer Research Center (DKFZ), Partner Site Tübingen, 72076 Tübingen, Germany; 7 Inflammation and Cancer Group, Department of Immunology and Microbiology, University of Copenhagen, 2100 Copenhagen, Denmark

**Keywords:** cancer vaccine, RhoC, CD4^+^ T cells

## Abstract

**Background**: A previous phase I/II study demonstrated potent and long-term immune responses in men with prostate cancer following vaccination with a 20mer synthetic peptide (RV001) derived from the Ras homolog gene family member C protein (RhoC). Moreover, a fraction of patients experienced prostate-specific antigen (PSA) responses, which prompted the initiation of a phase II double-blind randomized trial (NCT04114825). The primary endpoint was to study whether vaccination could postpone PSA progression. Furthermore, the study included an evaluation of vaccination-induced immune responses, and in-depth in vitro studies of RhoC-specific CD4^+^ T cell responses. **Methods**: Men with non-metastatic biochemical recurrence after either radical prostatectomy or radiation therapy were eligible for the study. Participants were randomized 1:1 to either subcutaneous injections of 0.1 mg/mL RV001 emulsified in Montanide ISA 51, or a placebo. Vaccinations were administered every 2 weeks for the first six times, then five times every 4 weeks for a total treatment time of 30 weeks. Blood samples were collected from a subset of patients (n = 38) over the course of vaccination, and peripheral blood mononuclear cells (PBMCs) isolated for immunological assessment of vaccine-induced immune responses. Experiments using PBMCs from a healthy donor and a patient were performed to study the phenotype and function of RV001-specific CD4^+^ T cells. **Results**: A total of 192 men entered the study. There was no difference in time to PSA doubling, with 7.5 versus 9.3 months, or in time to initiating further therapies, 11.2 versus 17.6 months for treatment and control groups, respectively. At long-term follow-up, 12.9% of the patients in the vaccination arm had developed metastasis compared to 12% in the placebo arm. No serious treatment-related side effects were observed, and treatment-related adverse events did not differ between groups. Immunological examinations in a subset of patients demonstrated that the vaccination induced potent, long-lasting CD4^+^ T cell responses capable of proliferation and cytokine production. RV001-specific CD4^+^ T cells were shown to mediate cytotoxicity against a RhoC-expressing cancer cell line in an HLA-class II-dependent manner. **Conclusions**: Men randomized to active treatment with RV001V demonstrated the induction of potent, functionally capable, anti RhoC-CD4^+^ T cell responses. However, there was no benefit in time to biochemical progression, and no difference in time to the initiation of second-line therapies.

## 1. Introduction

Following prostate cancer treatment with curative intent, some men will experience biochemical progression, thus facing additional therapies, either salvage procedures, or systemic treatment such as chemo- or endocrine therapy. Since subsequent therapies have side effects, including impaired sexual function and increased risk of developing comorbidities associated to long-term endocrine manipulations, there is an incentive to focus on developing agents that could prevent or delay cancer progression. In recent years, immunotherapies against prostate cancer with the ability to enhance the patient’s own immune response have been tested in several clinical trials [1]. Indeed, several prostate-associated or -specific T cell antigens have been described, and prostate cancer has been demonstrated to be sensitive to immunological targeting. In 2010, the Food and Drug Administration (FDA) approved Sipuleucel-T, a cell-manufactured cancer vaccine, based on improved overall survival [1]. More efficient therapies would, however, still be desirable, as would less complicated treatments with similar or better efficacy. Cancer vaccines in the form of synthetic peptides derived from cancer-associated or cancer-specific antigens represent a simpler and more applicable strategy to induce or boost anticancer T cell responses. Several studies conducted in patients with biochemical relapse or metastatic prostate carcinoma have indeed reported frequent induction of immune responses against vaccine peptides and clinical benefit in some patients [2,3,4].

The Ras homolog gene family member C (RhoC) is a small GTPase which has a limited expression in normal cells but high expression in advanced cancer cells and metastases [5]. RhoC plays a central role in cell migration and its role in cancer metastasis has been reported in several malignancies, including breast, gastric, colon, bladder, prostate, lung, pancreatic, and liver cancers [6,7,8,9], and, therefore, represents an attractive target for anticancer vaccination. We have reported that RhoC contains an HLA-A*03-binding CD8^+^ T cell epitope [10], and that an extended 20mer-long version of this peptide (RV001) is highly immunogenic in prostatectomized patients without progression (RhoVac-001 Phase I/II study, NCT03199872) [11]. Herein, we present the results from the double-blind, placebo randomized RhoVac-002 phase II study (NCT04114825) conducted to test whether vaccination with the RV001 vaccine (RV001V) could postpone biochemical progression in men with biochemical relapse following either radical prostatectomy or radiation therapy. Progression was assessed by the doubling time of the serum marker prostate-specific antigen (PSA). In a subgroup of patients, we conducted a thorough immunological assessment of vaccination-induced T cell responses, and of RhoC-specific CD4^+^ T cell reactivity based on in vitro established RV001-specific T cell lines and clones.

## 2. Materials and Methods

### 2.1. Study Design

**Figure 1:**
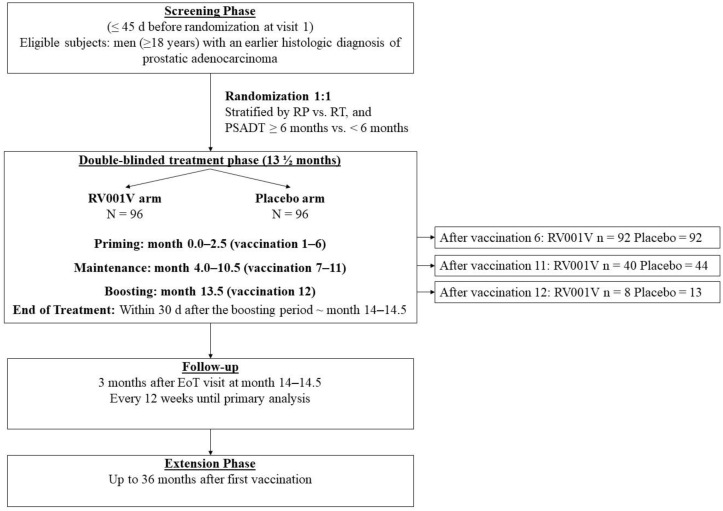
Outline of the study flow. EoT, end of treatment; PSADT, PSA doubling time; RP, radical prostatectomy; RT, radiation therapy.

This study was a phase II, double-blind, and randomized study (EudraCT Number 2019-000951-14) conducted at 35 centers in 7 countries. The primary endpoint was to investigate whether a vaccination regimen with multiple subcutaneous (SC) administrations of RV001 peptide vaccine (RV001V) could reduce PSA progression in men with biochemical relapse following curatively intended therapy for localized prostate cancer. Progression was assessed by the prostate-specific antigen doubling time (PSADT). Secondary endpoints included an evaluation of the safety and tolerability of RV001V following SC administration, time to subsequent antineoplastic therapy initiation, and evaluation of immune response in a subset of patients. The study flowchart is presented in Figure 1. The study consisted of a screening period ≤ 45 d before randomization at first visit, a double-blind treatment phase of 13½ months, and a follow-up phase 12 weeks after the end of treatment visit (EoT), and then continued follow-up every 12 weeks until the patient reached the primary endpoint or until the primary analysis was conducted. RV001V and placebo were both administered by SC injection in the upper arm alternating between the right and left side during a 12-dose regimen using a 1 mL syringe. The first 6 vaccinations (priming period) were given every 2 weeks, the following 5 vaccinations (from the 7th until the 11th) were administered with 4 weeks between each vaccination (maintenance period), and the last vaccination (the 12th) was administered 6 months following the 11th vaccination (boosting injection). The vaccination schedule is shown in Figure S1. Patients were randomized 1:1 using the interactive web response system (IWRS) which allocated an investigational medicinal product (IMP) kit containing either RV001V (0.1 mg/mL) in a solution with a pH of 3.5, or a placebo containing a sterile acetate buffered saline with the same pH. The peptide or placebo solution was emulsified in Montanide ISA-51 in a 50/50 ratio. Montanide ISA 51 is a commonly used adjuvant in peptide vaccination, which facilitates slow antigen exposure and a depot effect at the vaccination site [12].

Investigators, patients, and all study staff with direct patient contact were blinded. Patients progressing following radical prostatectomy with or without subsequent salvage radiation were eligible if PSA was > 0.2 ng/mL and PSADT ranged from 3 to 12 months. Patients previously treated with radiation were eligible if PSA was above nadir + 2 ng/mL and PSADT ranged from 3 to 12 months. Patients underwent standard imaging studies, including CT and bone scan, or PET-CT, to exclude metastatic disease prior to enrollment. For all inclusion and exclusion criteria, see Table S1. Patients were stratified by prior treatment and PSADT below or above 6 months prior to randomization, and sample size was calculated based on time to PSA progression. Patient characteristics are presented in Table 1. A subset of patients underwent extended immunological examination. In total, 38 patients (28 on active treatment and 10 from the placebo arm) were subjected to immunological monitoring of vaccine-induced immune reactivity as described in more detail below.

Patients were followed in out-patient clinics with repeated PSA measurements until meeting the predefined threshold for biochemical progression, PSA doubling compared to entry. When reaching the predefined endpoint, patients had an EoT visit within 30 d, and were followed in out-patient clinics every 12 weeks to record subsequent treatments, progression, and vital status. When reaching the primary endpoint, further treatment options were to be decided based on the clinical evaluation. Hypothesis testing to compare the survival distributions of the two groups was performed via a stratified log-rank test adjusting for stratification factors in the randomization. In addition, a Cox regression model adjusted by stratification factors was used to estimate the hazard ratio (HR) and its associated 95% confidence interval.

### 2.2. Isolation of Peripheral Blood Mononuclear Cells from Patients

Peripheral blood mononuclear cells (PBMCs) were isolated at visits 1 (n = 4) or 2 (pre-vaccination; priming period), visit 8 (four weeks after the 6th vaccination; maintenance period), and, if available, at visit 13 (3 months after the 11th vaccination; boosting period) from 100 mL heparinized blood within 8 h after blood drawing either at DanTrials Aps, Denmark, or at the Institute for Immunology, Tübingen, Germany. The blood was diluted 1:2 in Dulbecco’s phosphate-buffered saline (PBS, BioNordika, Herlev, Denmark) before PBMCs were isolated by density centrifugation using pre-filled 50 mL Leucosep tubes (Greiner Bio-One, Kremsmünster, Austria) according to a standard protocol. Cells were counted either with Trypan Blue and Tuerks solution (both Sigma-Aldrich, Burlington, MA, USA) or with the NucleoCounter^®^ NC-250TM (Chemometec, Allerød, Denmark). Seven to thirteen million (Mio) cells were frozen per cryovial (Nunc, Sigma-Aldrich) in 1 mL heat-inactivated (h.i.) fetal bovine serum (FBS, Life Technologies, Carlsbad, CA, USA) containing 10% dimethylsulfoxide (DMSO, Saveen Werner, Limham, Sweden). Cells were kept in freezing containers (Nalgene Mr. Frosty, Sigma-Aldrich) at −70/−80 °C and transferred within 3 d to liquid nitrogen (−196 °C). PBMC samples from DanTrials ApS, Copenhagen, Denmark, were sent on temperature-controlled dry ice to the Institute for Immunology of Tübingen, Germany, for immunological analysis. Cells were stored again in liquid nitrogen (−196 °C) up to 26 months before testing.

### 2.3. Isolation of Peripheral Blood Mononuclear Cells from Healthy Donor Blood

In total, 100 mL of blood from a healthy donor was collected in heparin tubes. PBMCs were isolated according to a standard, pre-established protocol using LymphoprepTM density gradient medium (Alere Technologies, Jena, Germany). Cells were counted with Trypan Blue (Sigma-Aldrich) using a counting chamber. Cells were used immediately or cryopreserved. For cryopreservation, h.i. FBS (Gibco, Grand Island, NY, USA) with 10% DMSO (Honeywell, Charlotte, NC, USA) was used. Freezing ampules were stored at −80 °C before transferring to −150 °C for long-term storage. For some experiments, PBMCs were isolated from healthy donor buffy coats following the same protocol.

### 2.4. Culturing of the FM3 Cancer Cell Line

The human melanoma cell line FM3 was used for co-culture experiments. Cells were thawed in prewarmed RPMI medium (Gibco). After two washes, cells were counted, and 3–5 Mio cells were resuspended in R10 medium (RPMI + 10% h.i. FBS) and transferred to a T25 tissue culture flask (Corning Incorporated, Corning, NY, USA). Cells were cultured in a humidified 37 °C, 5% CO_2_ incubator. Cells were checked for confluency and split if needed every second day. To do so, media was discarded and PBS (Sigma-Aldrich) was added to wash the cells. To dissociate the adherent cells from the flask, cells were incubated for 2–5 min at 37 °C in the presence of 0.5 mL trypsin + 0.25% ethylenediaminetetraacetic acid (trypsin-EDTA, Gibco). After dissociation, R10 medium was added and cells were split or transferred to a T75 tissue culture flask (Corning Incorporated) with fresh medium.

### 2.5. In Vitro Immunomonitoring for Anti-Vaccine T Cells

In total, and based on the previously demonstrated excellent immunogenicity of RV001V [11], only a subgroup of patients was subjected to immunological analysis, i.e., n = 28 patients on active treatment and n = 10 patients on placebo treatment with PBMCs collected at a pre-vaccination visit and visit 8 during vaccination. Available PBMCs obtained at visit 13 were also tested (n = 8 and n = 7 for the active and placebo groups, respectively). IFN-γ ELISpot testing and analysis was performed in a blinded fashion, either ex vivo after PBMC thawing or after in vitro cell expansion (IVASS). For the ex vivo ELISpot, cells were thawed and rested overnight in a T cell medium (IMDM) (Gibco) with 10% h.i. human serum (HS, Sigma-Aldrich), penicillin 100 units/mL and streptomycin 0.1 mg/mL (Sigma-Aldrich), 50 µM β-mercaptoethanol (Merck, Darmstadt, Germany), and 1 µg/mL DNAse I (Sigma-Aldrich). For the in vitro culture, PBMCs were thawed and cultured in the presence of the vaccine peptide (10 µg/mL), poly ICLC (20 µg/mL, Oncovir Inc., Washington, DC, USA), and interleukin-2 (rhIL-2) (R&D Systems, Minneapolis, MN, USA) in 48-well or 24-well plates for 12 d as described in detail elsewhere [11]. For the ELISpot, PBMCs were washed, counted, and stimulated in triplicate with 50 µg/mL RV001 peptide (1 mg/mL stock in sterile LiChrosolv water, Merck), the respective amount of LiChrosolv water (negative control, two-6 wells), or 10 µg/mL phytohemagglutinin-L (PHA) (positive control, Sigma-Aldrich). For the ex vivo and IVASS ELISpots, 0.4 and 0.2 Mio cells/well were plated, respectively. The ELISpot plate was incubated for 26 h at 37 °C, 7.5% CO_2_ before development. The detailed protocol has been published [11]. Wells with overdeveloped areas or with spot counts too numerous to count (TNTC) were set to a default spot count of 2000 spots.

For statistical response analysis, the distribution-free resampling method DFR(2x) was used [13]. Significant *p*-values are provided as follows: * *p* < 0.05; ** *p* < 0.01; *** *p* < 0.001. In addition, a threshold of ≥10 spots/well was applied for the ex vivo ELISpot. A patient was stated as an immunological responder based on the results of the post-IVASS ELISpot test at visit 8. If a T cell response was detected against RV001 at visit 1 or 2 (pre-vaccination), the specific T cell response at visit 8 after IVASS needed to be boosted at least 2-fold as compared to that of visit 2.

### 2.6. Establishment of RV001-Specific T Cell Cultures from a Healthy Donor

Dendritic cells (DCs) were generated from fresh PBMCs on day 0 and 7. CD14^−^ based magnetic sorting of monocytes was performed following the manufacturer’s instructions (Miltenyi Biotec, Bergisch Gladbach, Germany). CD14^+^ cells were cultured in 6-well plates in RPMI medium supplemented with 10% h.i. FBS in the presence of IL-4 (250 U/mL) and GM-CSF (1000 U/mL) (both PeproTech, Cranbury, NJ, USA). On day 5, DCs were matured by the addition of IL-1β (1000 U/mL), IL-6 (1000 U/mL), TNF-α (1000 U/mL) (all PeproTech), and prostaglandin E2 (PGE2) (1 µg/mL) (Sigma-Aldrich). On day 6, DCs were harvested and washed twice in serum-free X-VIVO 15 medium (Lonza, Basel, Switzerland). The RV001 peptide was dissolved in distilled H_2_O to a final concentration of 1 mg/mL. For sterile filtration, the solution was run through a Millex-GV filter with 0.22 μm pore size (Merck). Peptide aliquots were kept at 2–4 °C for a maximum of 7 d. For pulsing, 2 Mio cells were added per well in a 24-well plate and the RV001 peptide was added to a final concentration of 10 µM. For the 1st (day 7) and 2nd (day 15) RV001-specific T cell stimulations, pulsed DCs were harvested, washed twice, and resuspended in X-VIVO + 5% h.i. HS (Sigma-Aldrich) for a concentration of 3 Mio cells/mL. Freshly isolated CD14^−^ cells on day 7, containing T cells, were resuspended in X-VIVO + 5% h.i. HS for a concentration of 3 Mio cells/mL and co-cultured with pulsed DCs at a 10:1 ratio in a 24-well plate. Finally, 40 U/mL IL-7 was added the same day and 20 U/mL IL-12 was added the following day (both PeproTech). For the 3rd (day 22), 4th (day 31), and 5th (day 39) stimulations, irradiated peptide-pulsed autologous PBMCs were used at a 1:1 ratio. The following day, 120 U/mL human interleukin (IL-2) (PeproTech) was added to each well.

### 2.7. Establishment of RV001-Specific T Cell Cultures from a Patient Blood Sample

For the establishment of RV001-specific cultures, cells of a vaccinated patient (patient 101-003) were used. Patient 101-003 was selected as he had readily developed RV001-specific T cells post vaccination. PBMCs from visit 8 were thawed in X-VIVO 15 containing 5% h.i. HS. After one washing step with serum-free medium, PBMCs were counted, seeded at 1.0–3.5 Mio or 3.5–6.5 Mio cells/well in X-VIVO + 5% h.i. HS in a 48-well or 24-well plate, respectively, and further cultured at 37 °C and 5% CO_2_ (day 0). For the IVASS, cells were stimulated with 10 µg/mL RV001 peptide plus 20 µg/mL poly ICLC (Hiltonol, Oncovir) on day 1. IL-2 (PeproTech) was added to the culture at 2 ng/mL on days 3, 5, 7, and 9. On day 12, cells were harvested, counted, and either enriched for RV001-specific T cells or tested with the intracellular cytokine staining (ICS) assay. For the schematic protocol and gating strategy, see Figure S2.

### 2.8. Enrichment and Sorting of RV001-Specific T Cell Cultures and T Cell Cloning

After five peptide stimulations for the priming of healthy donor (HD) T cells, or after 12 d of in vitro stimulation for the IVASS of patient T cells, T cells were co-cultured with peptide-pulsed FM3 cells for 4 h at 37 °C and 5% CO_2_, and subsequently enriched based on their TNF-α secretion using the TNF-α secretion assay—cell enrichment and detection kit (Miltenyi Biotec) following the manufacturer’s instructions. For peptide pulsing, FM3 cells were incubated with 10 µM RV001 for 2 h and subsequently washed with RPMI. Sorted cells were plated at 0.3, 1, or 3 cells per well in a 96-well plate in the presence of 100 µL cloning mix. For the cloning mix preparation, 30 Mio PBMCs from 3 different healthy donors were thawed and irradiated with 25 GY in a GammaCell irradiator (Best Theratonics Ltd., Ottawa, ON, Canada). After irradiation, feeder cells were incubated for 2 h at 37 °C and 5% CO_2_ in the presence of phytohaemaglutinin P (PHA-P, Sigma-Aldrich) at 1 µg/mL. IL-2 (PeproTech) was added to the cells to a final concentration of 100 U/mL. Twice a week, 25 µL fresh medium + 5% h.i. HS with IL-2 was added to each well. Peptide-specific clones were selected based on the IFN-γ ELISpot and expanded following a rapid expansion protocol (REP). For each REP culture, 20 Mio feeder cells from 3 different healthy donors were resuspended in 20 mL medium + 5% h.i. HS and incubated for 1–2 h at 37 °C, 5% CO_2_. After incubation, the feeder cells were transferred to a T25 flask together with the clone in the presence of 6000 U/mL IL-2, 0.6 µg anti-CD3 antibody (eBioscience, San Diego, CA, USA; clone OKT3), and 0.5 mL HEPES (Life Technologies), and cultured at 37 °C, 5% CO_2_. After 5 d of culture, 10 mL of medium was replaced with fresh medium + 5% h.i. HS + 6000 U/mL IL-2. This was repeated twice a week until the culture started expanding. Thereafter, the culture was counted and adjusted to 0.5 Mio cells/mL.

### 2.9. Intracellular Cytokine Staining

The ICS assay was used to assess the production of cytotoxic molecules and degranulation markers by T cell lines or clones upon RV001 peptide stimulation. For that, 0.3 Mio T cells were added to each well in a 96-well plate in the presence of different stimuli. Experiments were performed using a single well per condition due to the limited availability of cells. For short peptide stimulation, a final concentration of 5 µM per well was used. As a positive control, 25 ng/mL phorbol 12-myristate 13-acetate (PMA, Sigma-Aldrich) and 375 nM ionomycin calcium salt (Sigma-Aldrich) were used. For background measurement, medium alone was added to the wells. Finally, 0.3 µL of phycoerythrin (PE)-conjugated anti-CD107a antibody (BD, Franklin Lakes, NJ, USA) was added to each well, and cells (final volume 200 µL) were incubated at 37 °C, 5% CO_2_. After 1 h, 2 µL of Brefeldin A (BioLegend, San Diego, CA, USA) was added for a final concentration of 5 µg/mL and cells were incubated for 4 more h before staining. Afterwards, cells were washed twice with 150 µL/well of fluorescence-activated cell sorting (FACS) buffer (PBS + 10% FBS). Cells were then stained with cell surface antibodies (see Table S2) and incubated for 30 min at 4 °C in the dark. To fix and permeabilize the cells, the fixation/permeabilization concentrate and the diluent (both Invitrogen, Carlsbad, CA, USA) were combined in a 1:4 proportion and 200 µL/well was added as per manufacturer’s instructions. Cells were left at 4 °C overnight. The following day, cells were washed twice with 150 µL permeabilization buffer (Invitrogen) diluted 1:10 in sterile H_2_O, and stained with the intracellular antibodies for 30 min at 4 °C in the dark. Finally, cells were washed twice in permeabilization buffer and resuspended in 120 µL FACS buffer. A total of 110 µL of each sample was acquired using a NovoCyte Quanteon flow cytometer (Agilent, Santa Clara, CA, USA). Analysis was performed with NovoExpress (V.1.5.0). For the gating strategy, see Figure S2.

### 2.10. T-Cell Receptor Beta-Chain Variable Region Flow Cytometry Panel

The IOTest Beta Mark kit (Beckman Coulter, Brea, CA, USA) is designed for quantitative determination of the T-cell receptor beta-chain variable region (TCRBV) repertoire of human T lymphocytes by flow cytometry. The kit contains 24 different specificities, covering about 70% of the normal human TCRBV repertoire. Peptide-specific T cell lines or clones were harvested, counted, and transferred to eight tubes (approximately 0.3 Mio cells/tube). Cells were resuspended in FACS buffer and centrifuged at 1500 rpm for 5 min twice. Cells were stained with antibodies for 20 min at 4 °C in the dark. Then, cells were washed before acquisition on the NovoCyte Quanteon flow cytometer. Analysis was performed with NovoExpress (V.1.5.0). For the gating strategy, see Figure S3.

### 2.11. T-Cell Receptor (TCR) PCR Panel

RNA was isolated with the NucleoSpin RNA kit (Macherey-Nagel, Düren, Germany) and reverse-transcribed using the SuperScript VILO cDNA Synthesis Kit (Invitrogen). cDNA was tested and pooled (9 clones), and amplified using primers specific for TCRBV regions 1–24 together with a constant region primer as described [14]. In total, 10 uL aliquots were electrophoresed in a 2% agarose gel together with a 100 bp ladder (Thermo Fisher Scientific, Waltham, MA, USA), and stained with SYBR green (Applied Biosystems, Waltham, MA, USA) under UV light.

### 2.12. Western Blotting

Briefly, FM3 cells were lysed using RIPA lysis buffer (Thermo Fisher Scientific, cat. 89900) supplemented with a protease and phosphatase inhibitor cocktail (Thermo Fisher Scientific, cat. 78420). Proteins were quantified by BCA assay (Pierce, Rockford, IL, USA) and separated using precast 4 to 12% Bolt Bis-Tris Plus SDS-PAGE gels (Invitrogen). Proteins were transferred to nitrocellulose membranes (Thermo Fisher Scientific) using the iBlot 2 system (Invitrogen). For RhoC protein detection, the rabbit anti-human RhoC antibody (Cell Signaling Technology, Danvers, MA, USA) was used at the manufacturer’s recommended concentration. Proteins were visualized using the SuperSignal West ECL Kit (GE Healthcare, Chicago, IL, USA) and Bio-Rad ChemiDoc Molecular Imager (Bio-Rad, Hercules, CA, USA). Quantification of the signal was performed using Fiji ImageJ (v.1.49).

### 2.13. xCELLigence Real-Time Cell Analysis Assay

The xCELLigence system was used to assess the cytotoxicity of peptide-specific T cell lines or clones against peptide-pulsed or unpulsed FM3 cancer cells. First, 100 µL/well RPMI was added to the E-plate 96 (ACEA Biosciences, San Diego, CA, USA), which was introduced in the xCELLigence SP system (ACEA Biosciences) to measure the background signal. FM3 cells were counted and seeded at a density of 5000 cells/well. After 30 min on the bench, the plate was re-introduced in the xCELLigence SP system and incubated for 24 to 48 h. When the cell index was approximately 1, the RV001 peptide (10 µM) was added for pulsing the FM3 cells. After 2 h, the medium was removed and T cells were added at different effector-to-target ratios (E:T) = 10:1, 3:1, and 1:1. As a negative control, some wells were left with target cells only. The final volume was 200 µL. After the addition of T cells, the plate was re-introduced into the xCELLigence SP system and the readout was resumed. Impedance was measured at varying intervals for the following 72 h. Acquired data were analyzed with the xCELLigence RTCA Software Pro 2.6.0 (ACEA Biosciences). All data points were normalized to the point of T cell addition (Figures S5 and S6). All conditions were performed in triplicate; the sample mean and standard deviation were calculated and plotted in GraphPad Prism 8 (GraphPad Software, Boston, MA, USA).

## 3. Results

### 3.1. Clinical Outcome

Patient characteristics were well balanced between treatment arms; approximately 75% of the patients had Gleason Group 3 or higher, at either final histo-pathology or biopsies prior to radiation, which indicates moderate to high aggressiveness of prostate cancer according to the standard classification system (the flow chart of the study is presented in Figure 1 and patient details are in Table 1).

**Figure 2:**
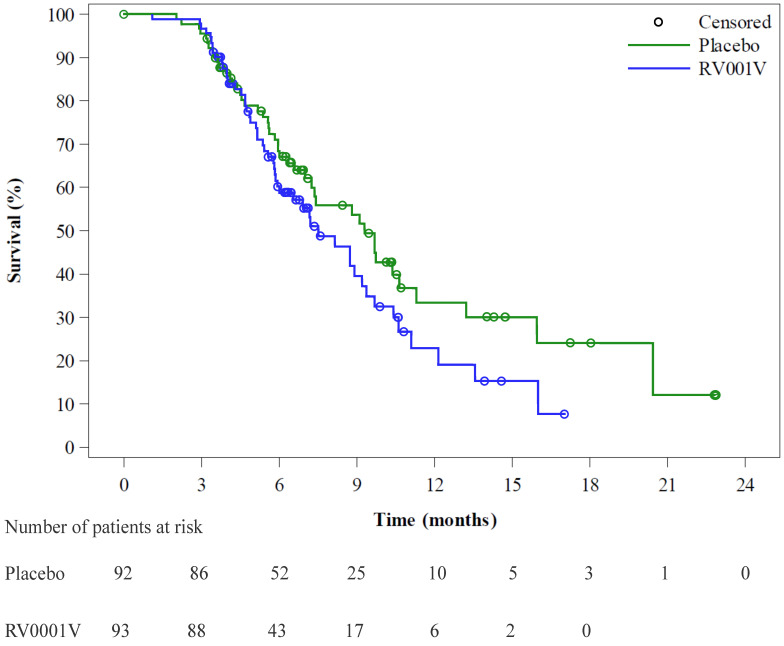
Time to PSA doubling compared to baseline PSA at study entry. *p* = 0.1327.

Patients were followed for biochemical progression with PSA measured at each out-patient clinic visit during the period studied. In total, 42 patients (45.2%) in the experimental arm and 41 (44.6%) in the control group experienced PSA doubling compared to baseline PSA, with a median time to PSA doubling of 7.5 (0.9–9.2) and 9.3 (7.2–11.3) months, respectively, and the time to PSA doubling being non-significantly different between the two groups (Figure 2). Furthermore, no differences in time to subsequent treatments, in clinical progression-free survival, or in survival were recorded. Safety and tolerability were evaluated by the frequency and severity of treatment-related adverse events. Overall, 171 of 196 (87.2%) of the participants reported one or more adverse events during follow-up, with no difference between the two groups 90.6% and 87.5%, for the active vs. placebo cohorts, respectively. The most frequently reported side effect was a local reaction at the injection site (155 participants). Serious adverse events were reported by seven (active cohort) and five (placebo cohort) participants. During the study period, four deaths occurred, one prior to randomization and three during treatment or follow-up, none of these related to the study vaccination.

### 3.2. Vaccination Against RhoC Induces Potent and Lasting Vaccine-Specific T Cell Responses

**Figure 3:**
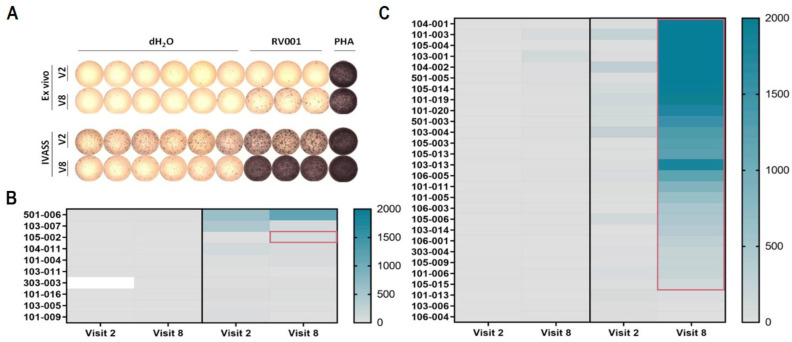
The majority of patients treated with RV001V develop an immunological response against the vaccine. T cell reactivity against the vaccine peptide (RV001) was tested in IFN-γ ELISpot ex vivo and after in vitro pre-stimulation (IVASS). (**A**) Exemplary ELISpot plate for patient 104-002 at visits 2 (V2, pre-vaccination) and 8 (V8, after the 6th vaccination) ex vivo (**upper panel**) or after IVASS (**lower panel**). (**B**,**C**) Heat-maps showing the specific mean spot counts (background subtracted) for each tested patient at visits 2 and 8 for the placebo treatment group ((**B**) saline + Montanide, n = 10) and the active treatment group ((**C**) RV001 + Montanide, n = 28). The scale represents the mean number of specific spots per well. Ex vivo and post-IVASS settings are shown in the left and right panels, respectively. The white rectangle indicates that the ELISpot test was not performed. Red frames indicate immunological responders according to the predefined criteria.

IFN-γ ELISpot testing was performed either ex vivo after PBMC thawing or after IVASS. ELISpot results are summarized for all n = 38 tested patients in Figure 3 for pre- (visit 1 or 2) and post-vaccination (visit 8) timepoints. Exemplary ELISpot wells are shown for the ex vivo and IVASS settings in Figure 3A (patient 104-002 of the active treatment group, visits 2 and 8). The vaccine-specific spot counts are displayed as heat-maps for the placebo-controlled group (saline + Montanide, n = 10 patients, Figure 3B) and the active treatment group (RV001V + Montanide, n = 28 patients, Figure 3C), and mean specific spots counts and analysis of responses are displayed in Tables S3–S6. In total, anti-vaccine T cells were detected in 8/38 (21.0%) and 26/38 (68.4%) patients in the ex vivo (Figure 3B,C left panels) and IVASS (Figure 3B,C right panels) ELISpot, respectively. All patients exhibiting an ex vivo anti-vaccine response belonged to the active treatment group (8/28; 28.5%). Of the 26 patients whose PBMCs responded after IVASS, 25 patients were under active treatment (25/28; 89.3%), whereas only one patient (patient 105-002) had received the placebo injection (1/10; 10.0%). Anti-vaccine T cell reactivity in this patient was only transiently detected at visit 8 but not anymore after vaccination termination (visit 13). In contrast, patients on active treatment with a vaccine-specific response at visit 8 were still responders at visit 13 (n = 6 patients tested, Table S4). One tested patient from the placebo-controlled group also showed an anti-RV001 response only at visit 13. Altogether, RV001V was immunogenic in the majority (89.3%) of the patients on active treatment. This is very similar to the frequencies we found in our previous phase I/II study (86% of responding patients) [11]. Based on this earlier study, we expect that the majority of these responses were mediated by CD4^+^ T cells.

### 3.3. A CD4^+^ T Cell Clone Mediates RhoC-Specific Cancer Cell Killing

**Figure 4:**
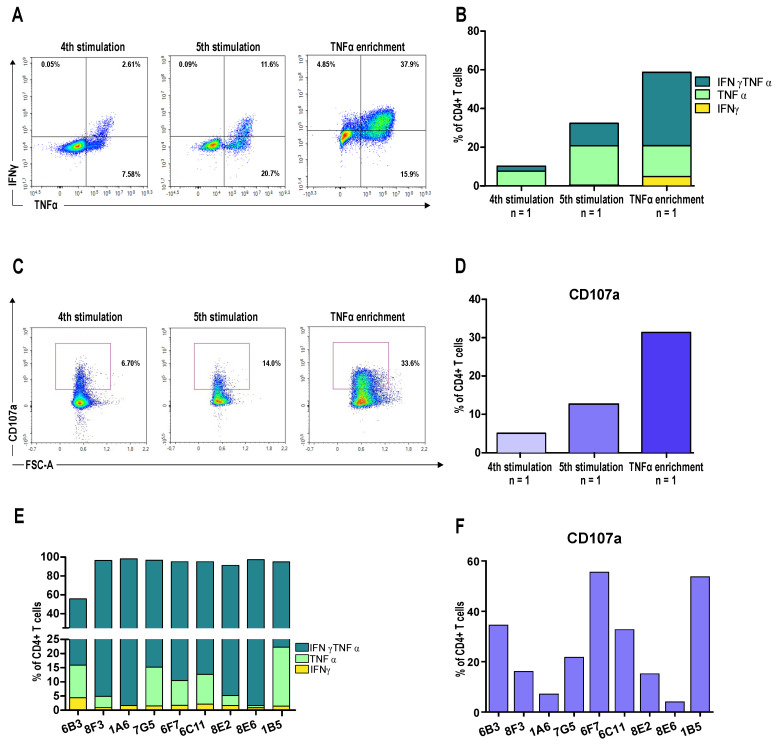
CD4^+^ RV001-specific T cell responses observed in HD2 upon stimulation with RV001. CD14^−^ cells from HD2 were stimulated with RV001-pulsed DCs or PBMCs for two and three rounds, respectively, and specific T cells were enriched based on TNFα secretion. Peptide-specific reactivity was assessed within the CD4^+^ subset using the ICS assay. (**A**) Flow cytometry dot plots demonstrating TNFα and IFNγ production in CD4^+^ T cells after the 4th and 5th stimulations and after enrichment. (**B**) Corresponding stacked bar charts illustrating the percentage of IFNγ^+^, TNFα^+^, and TNFα^+^/IFNγ^+^ CD4^+^ T cells when stimulated with RV001, after subtraction of the background. (**C**) Flow cytometry dot plots showing the expression of the cell surface degranulation marker CD107a on gated CD4^+^ T cells. FSC-A, forward scatter. (**D**) Corresponding bar plot illustrating the percentage of CD107a^+^ CD4^+^ T cells when stimulated with RV001 after subtraction of the background. (**E**) Stacked bars summarizing the percentage of IFNγ^+^, TNFα^+^, and TNFα^+^/IFNγ^+^ CD4^+^ T cells for each of the selected clones upon peptide restimulation. (**F**) Bar plot of the percentage of CD107a^+^ CD4^+^ T cells of the selected clones upon peptide restimulation. For all panels n = 1.

We sought to mimic vaccination by generating a CD4^+^ RV001-specific T cell culture from a healthy donor (HD2). To do so, we performed two rounds of stimulation with peptide-loaded autologous DCs and three rounds of stimulation with peptide-loaded PBMCs. The specificity of the culture was tested with intracellular cytokine staining after the fourth and fifth stimulations. The percentage of CD4^+^ T cells producing TNFα alone increased from 7.58% to 20.7%, CD4^+^ T cells producing IFNγ alone went from 0.05% to 0.09%, and CD4^+^ T cells producing both cytokines increased from 2.61% to 11.6% after the fourth and fifth stimulation, respectively (Figure 4A,B). Cell membrane expression of the degranulation marker CD107a followed the same trend (Figure 4C,D). After five stimulations, T cells were enriched based on TNFα production, resulting in 15.9% of CD4^+^ cells producing TNFα alone, 4.85% producing IFNγ alone, and 37.9% producing both cytokines (Figure 4A,B, right panels). After enrichment, cells were cloned, and the expanded clones were first screened for RV001 reactivity using the IFNγ ELISpot (Figure S4). Selected CD4^+^ T cell clones (n = 9) were expanded if needed and assessed for cytokine production with intracellular staining.

**Figure 5:**
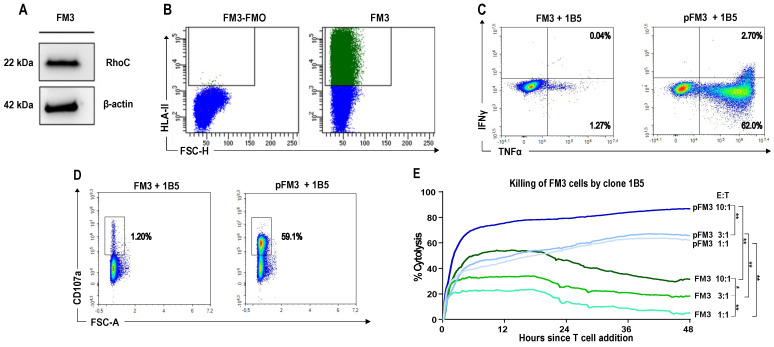
Primed T cells from a healthy donor can mediate cytotoxicity against RhoC- and HLA-II-expressing FM3 cancer cells. (**A**) Western blot of FM3 to demonstrate RhoC expression. Full blots in Figure S7. (**B**) Flow cytometry of HLA-II expression in FM3 cancer cells using a pan-HLA-DR antibody. FMO, fluorescence minus one; FSC-H, forward scatter height. (**C**) Clone 1B5 was co-cultured with FM3 (**left**) and peptide-pulsed FM3 (pFM3) (**right**). Cytokine production was examined in living CD4^+^ T cells. Percentages of TNFα^+^ and IFNγ^+^ are given. (**D**) For the same co-culture experiment, surface expression of the degranulation marker CD107a is shown. FSC-A, forward scatter. (**E**) Cytotoxicity of clone 1B5 against FM3 (green) and peptide-pulsed FM3 (pFM3) (blue) was assessed with the xCELLigence system at different effector-to-target (E:T) ratios (10:1; 3:1; 1:1) over a total period of 48 h. Statistical significance was determined by two-way analysis of variance (ANOVA). * indicates *p* < 0.05 and ** indicates *p* < 0.01.

Clone 1B5, which showed high cytokine production and high CD107a expression upon RV001 peptide restimulation (Figure 4E,F), was selected for further cytotoxicity assays. The human melanoma cell line FM3 naturally expresses high levels of the RhoC protein (Figure 5A) and of HLA-class II molecules (Figure 5B). We co-cultured the RV001-specific CD4^+^ T cell clone 1B5 with peptide-pulsed or unpulsed FM3 cells. Importantly, FM3 cells and HD2 shared HLA-II molecules DQB1 06:02, and 06:04, respectively (Table S7).

A small percentage of 1B5 cells, 1.31%, produced TNFα alone or in combination with IFNγ when co-cultured with unpulsed FM3 cancer cells, which increased to 64.7% when co-cultured with peptide-pulsed cancer cells (Figure 5C). This was also mirrored in the cell membrane expression of the degranulation marker CD107a, which increased from 1.20% to 59.1% in the presence of pulsed FM3 cells (Figure 5D). Next, we used the xCELLigence real-time cytotoxicity method to assess the killing potential of the 1B5 CD4^+^ T cell clone.

Killing of both pulsed and unpulsed cancer cells was seen (up to 87% and 53% cytolysis at 10:1 E:T ratio, respectively). Cytolysis was stronger with increasing effector-to-target ratios (Figure 5E).

### 3.4. Patient-Derived CD4^+^ T Cells Mediate Cancer Cell Killing

**Figure 6:**
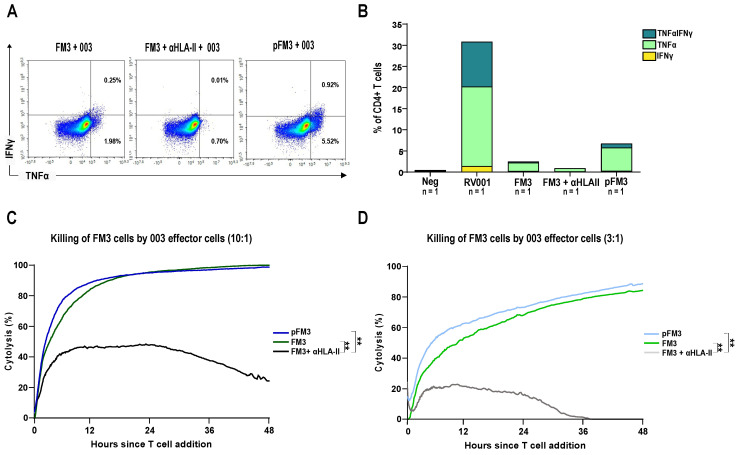
Patient-derived CD4+ T cells can mediate cytotoxicity against FM3 cancer cells. (**A**) Twelve day-cultured PBMCs from patient 101-003 (003) were co-cultured with FM3 (**left**), FM3 with a blocking antibody against HLA-II (**middle**), and peptide-pulsed FM3 (**right**). Cytokine production was examined in living CD4+ T cells. (**B**) Stacked bar charts illustrate the percentage of IFNγ+, TNFα+, and TNFα+/IFNγ+ CD4+ T cells of the same co-culture experiment. For all conditions, n = 1. (**C**,**D**) Cytotoxicity of the CD4+ T cell line against FM3 (green) and peptide-loaded FM3 (pFM3, blue) was assessed with the xCELLigence system at different effector-to-target (E:T) ratios (10:1 (**C**) and 3:1 (**D**)) over 48 h. Killing of FM3 in the presence of the HLA-II blocking antibody was also assessed (grey). Statistical significance was determined by two-way ANOVA (**C**,**D**). ** indicates *p* < 0.01.

Next, we assessed CD4^+^ T cells derived from the vaccinated patient 101-003 who was a strong responder to the vaccine (Figure 3A). This patient shares the same HLA-DPB1 and HLA-DQB1 molecules with FM3 cancer cells (both positive for DPB1 04:01, DQB1 06:03 and 06:02, respectively, Table S7). PBMCs obtained during vaccination (visit 8) were subjected to IVASS in the presence of the RV001 peptide. Following this 12 d stimulation, cells were co-cultured with peptide-pulsed FM3 cells, and TNFα-producing cells were enriched. After enrichment and expansion, cytokine production was re-assessed. Upon restimulation with the RV001 peptide, 18.8% of the CD4^+^ T cells produced TNFα alone, 1.42% produced IFNγ alone, and 10.6% produced both cytokines. Interestingly, 2.23% of CD4^+^ T cells also produced TNFα when co-cultured with unpulsed FM3 cells. When an HLA-II blocking antibody was added during the stimulation, TNFα-producing cells were reduced threefold down to 0.71%. As seen above with the clone 1B5, pulsed FM3 cells induced a higher production of TNFα, increasing to 6.44% (Figure 6A,B).

Next, cytotoxicity of the T cell line was assessed. Both peptide-pulsed and unpulsed FM3 cancer cells were efficiently killed at different E:T ratios. In accordance with the cytokine production, peptide-pulsed FM3 cells were killed faster than unpulsed cells, especially at the 3:1 ratio. Notably, the inclusion of an HLA-II blocking antibody yielded a substantial reduction in cytolysis, highlighting the dependency of this killing mechanism on HLA-II (Figure 6C,D).

### 3.5. Identification of TCRBV20

**Figure 7:**
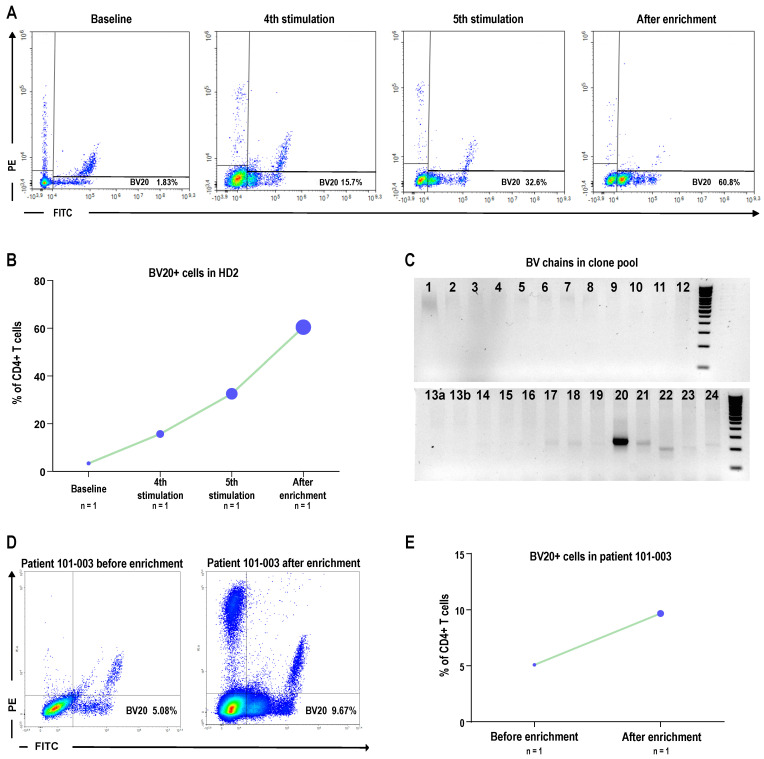
Preferential TCRBV usage in RV001-specific CD4^+^ T cells. (**A**) Dot plots gated on HD2 CD4^+^ T cells showing the population positive for BV20 in the lower right quadrant (single positives for fluorescein isothiocyanate, FITC), BV18 (single positives for phycoerythrin, PE), and BV5.1 (PE/FITC double positives). (**B**) Graphical representation of the percentage of CD4^+^ T cells positive for BV20 at baseline, after the 4th and 5th stimulations, and following TNFα enrichment. (**C**) PCR product from amplification of cDNA pooled from 9 clones electrophoresed in agarose gel and stained with SYBR green. Full blots in Figure S8. (**D**) Dot plots gated on live CD4^+^ T cells of patient 101-003 after IVASS and enrichment showing the population positive for BV20 in the lower right quadrant. (**E**) Graphical representation of the percentage of CD4^+^ T cells positive for BV20 in the same patient.

To characterize further the RV001-specific cytotoxic CD4^+^ T cells, we next studied the TCR beta variable (BV) chains of the healthy donor-derived clone 1B5 and of the patient-derived T cell line. Interestingly, we observed the expansion of a specific BV chain (BV20) throughout peptide stimulations during T cell priming (Figure 7A,B). Once clones were generated, we confirmed the monoclonal nature by PCR and, strikingly, all the clones shared the BV20 chain (Figure 7C). Subsequently, when generating the peptide-specific population derived from patient 101-003, we closely monitored the presence of BV20. Following enrichment, there was a notable increase in the percentage of BV20-expressing cells, from 5.08% to 9.67% of CD4^+^ T cells, suggesting a potential key role for T cells expressing this BV region in the recognition of RV001 (Figure 7D,E).

## 4. Discussion

In the original phase I/II clinical study, we demonstrated the safety and immunogenicity of the RV001V application. In addition, two patients experienced an apparent decrease in PSA, indicating that the immunological response may be coupled to the clinical control or delay of tumor progression [11]. These encouraging results prompted the initiation of a phase II randomized double-blind clinical study including an active treatment and a placebo control group. In line with previous results, the toxicity in the present phase II study was limited, and no difference was found between men receiving active treatment compared to a placebo.

A number of studies have tested various immunological approaches in the management of prostate cancer, which is a slowly growing cancer and could be an ideal target for immunotherapy. In fact, Sipuleucel-T, a prostate cancer vaccine, was approved by the FDA in 2010 for the treatment of metastatic, castration-resistant prostate cancer, i.e., late-stage disease patients, based on a marginal improvement in overall survival compared to a control [15]. Although the approval of the vaccine was controversial at the time, and the vaccine never reached widespread use, it does suggest that therapeutic vaccines in prostate cancer can delay disease progression.

Sipuleucel-T is a cell-based vaccine targeting the prostate antigen prostatic acid phosphatase, a differentiation antigen expressed in both normal and transformed prostate cells [15,16]. RhoC is a Rho GTPase and has been shown to play an important role in cell motility and metastasis formation. In addition, RhoC is absent or expressed at very low levels in normal non-dividing cells, but cancer cells express RhoC at high levels [6]. Thus, we envisioned that the targeting of RhoC in vaccination could lead to the selective eradication of prostate cancer cells with metastatic potential [11]. Moreover, despite the approval of Sipuleucel-T, a long list of vaccines has failed in phase III trials in spite of promising results in early-stage clinical testing [17]. Several reasons can be put forth to explain these failures, one of them being that many trials were conducted at a relatively late stage of disease. In this setting, vaccine-specific T cells not only need to target an excessive tumor mass but also to overcome the systemic immunosuppressive influence of the spreading cancer. For the current vaccine, we chose to vaccinate at an early stage of disease, with limited tumor mass, and supposedly a less suppressive impact systemically. The study included men having biochemical progression following curatively intended treatment and having biochemical progression combined with histo-pathological characteristics indicating an imminent risk of further progression. Thus, we selected a group of patients who would likely face endocrine treatment and in whom delaying time to further treatment is highly warranted. Moreover, to ensure a “minimal residual disease” status, patients with a PSA of 10 ng/mL or higher or positive for bone metastases were excluded. Despite the study being able to demonstrate a significant and long-lasting immunological response, there was no evidence for the clinical benefit of RV001V, either in terms of delaying further biochemical progression or in postponing time to further therapy.

Obviously, the first success criteria of a cancer vaccine is the induction of an immunological T-cell-mediated response. Early therapeutic cancer vaccination trials have focused on the induction of CD8^+^ T cell responses, for example, using minimal HLA-class I restricted peptides as antigens. Administration of such peptides along with adjuvants or even DCs did indeed induce detectable CD8^+^ T cell responses, although at a very low frequency. Importantly, when tested in phase III trials, clinical efficacy was not demonstrated [17]. One common explanation for the low frequency of vaccination-induced CD8^+^ T cells is an insufficient concomitant CD4^+^ T cell help. In addition, CD4^+^ T cells exert a range of direct and indirect functions that can contribute to tumor elimination [18]. This has encouraged the use of long synthetic peptides containing both HLA-class I and potentially HLA-class II epitopes that showed superiority in vaccination studies in mouse tumor models, and also, more recently, promising results in melanoma using lipoprotein signal peptidase (LSP) neo-peptide antigens. However, using this approach seems to mainly induce CD4^+^ T cells [18], as we also find in our immune monitoring, and to be less effective in inducing CD8^+^ T cell responses. To the latter, we have shown that the RV001 peptide comprises HLA-class I epitopes presented by HLA-A*03:01 and HLA-B*27:05 [11]. However, in our previous RhoC-vaccination study, we did not detect any CD8^+^ T cells restricted by HLA-A*03:01 (n = 2 HLA-A*03:01^+^ patients) but did detect a single CD8^+^ response restricted by HLA-B*27:05 (1 out of 3 B*27:05^+^ patients) [11]. In following experiments, we were able to identify one RV001-derived HLA-B*27:05 binding peptide by an immunopeptidomics approach. Regardless, clearly, the RhoC-specific response induced by our vaccine is largely constituted by CD4^+^ T cells.

Although the CD8^+^ T cell is the main cytotoxic effector cell in anti-virus and -cancer responses, CD4^+^ T cells can also kill [18,19]. Moreover, cancer cells, including prostate carcinoma, may express some level of HLA-class II molecules or upregulate HLA-class II molecules upon IFN-γ exposure [20]. Aiming to study the capacity of RhoC-specific CD4^+^ T cells to kill cancer cells, we generated RV001-specific CD4^+^ T cells both from a healthy donor (after in vitro priming with autologous DCs loaded with the RV001 peptide) and from a vaccinated patient (after short recall culture in the presence of RV001). CD4^+^ T cell lines and clones generated from these two donors were indeed capable of killing the melanoma cell line FM3 which expresses endogenously both HLA-class II and the RhoC protein. Moreover, the in vitro-generated clones showed the same cytokine production profile (TNFα and IFNγ) as well as cell membrane expression of the degranulation marker CD107a after stimulation, as we observed in vaccinated patients in the previous study [11]. Moreover, the TCRBV expression analysis shows that vaccine-specific CD4^+^ clones preferentially use one TCRBV region (BV20) both in the HD and the patient.

Therefore, the finding that the in vitro-generated CD4^+^ T cells are cytotoxic suggests that the vaccination-induced T cell response comprises CD4^+^ T cells with killing capacity towards RhoC, at least as judged by in vitro assays using class II-expressing cancer cells as targets. Whether this killing of cancer cells also takes place in vivo, however, remains unknown. On the one hand, the clinical data suggest that killing did not take place, at least not to a level that was capable of controlling or even delaying tumor progression. On another hand, the presence of RhoC-specific T cells before vaccination or in the placebo control group (Figure 3) strongly suggests that in vivo T cell priming was taking place in some patients; hence, that RhoC is a tumor antigen that can spontaneously give rise to T cell responses. To this end, it is noteworthy that FM3 is a cell line which expresses very high levels of HLA-II molecules, which may not be the case for the patients’ prostate cancer cells [21]. In addition, although RhoC is described as a widely expressed antigen, we cannot formally exclude that the early-stage disease patients did not express RhoC in their tumors.

Immunotherapy of cancer, e.g., the use of immune checkpoint inhibitors, has changed the outlook for cancer patients in a range of cancer indications [22]. However, prostate cancer is not sensitive to checkpoint inhibitor therapy, and once metastasized to distant sites, normally bone, prognosis is dismal [23]. Prostate cancer is rarely subjected to massive infiltration by cells of the immune system and is consequently denoted as a “cold” tumor, a tumor type which, opposed to the immune cell-infiltrated “hot tumors”, is less prone to responding to immune therapy [24]. Given the complexity of the immune microenvironment and the largely cold characteristics of prostate tumors, even the effective induction of vaccination-specific CD4^+^ T cells may have been insufficient to tip the balance in favor of a warmer microenvironment. Additionally, despite the generally effective induction of T cell responses, not all patients responded equally well, highlighting variations in immune system reactivity among patients. It could be speculated that vaccination could represent a means to convert the cold tumor by inducing anticancer T cell responses that may not possess stand-alone clinical efficacy but could condition the tumor for response to immune checkpoint inhibition. In our previous analyses, we found that vaccine-induced RhoC-specific CD4^+^ T cells expressed PD-1. Nonetheless, the induction of a more biased T cell response, i.e., both CD4^+^ and CD8^+^ T cell responses, would probably be more likely to lead to clinical efficacy. To this end, the 20mer synthetic RV001 peptide could be used as a “helper peptide” for combination with peptides with a known capacity to induce CD8^+^ T cell responses, and possibly in combination with checkpoint blockade. Alternatively, RV001V could be applied early on after curative treatment in high-risk patients, a timepoint where the development of metastases might be easier to counteract.

## 5. Conclusions

In conclusion, in this randomized study using PSA doubling time as a surrogate marker for cancer progression, we found no indication that the RhoC-targeting 20mer peptide vaccine was able to delay tumor progression measured as time to PSA doubling, despite inducing a robust CD4^+^, RhoC-specific immunological reaction. Since RhoC is one of the few antigens which has been shown to be expressed in metastatic disease, we propose that targeting this protein by vaccination should be combined, e.g., with checkpoint inhibitor therapy, to increase clinical efficacy.

## Figures and Tables

**Table 1:** Patient characteristics. ECOG, Eastern Cooperative Oncology Group; PSADT, PSA doubling time; SD, standard deviation.

	RV001V	Placebo	Overall
	(n = 96)	(n = 96)	(n = 192)
**Median age (range)**	70.5 (56–86)	70 (57–86)	70 (56–86)
Mean baseline PSA (ng/mL)	1.90 (2.09)	2.34 (2.35)	2.12 (2.23)
PSADT at randomization [n (%)]			
≥6 months	47 (49.0%)	52 (54.2%)	99 (51.6%)
<6 months	49 (51.0%)	44 (45.8%)	93 (48.4%)
Gleason Grade group [n (%)]			
Number	95	96	191
Grade 1	3 (3.2%)	6 (6.3%)	9 (4.7%)
Grade 2	14 (14.7%)	25 (26.0%)	39 (20.4%)
Grade 3	42 (44.2%)	28 (29.2%)	70 (36.6%)
Grade 4	13 (13.7%)	21 (21.9%)	34 (17.8%)
Grade 5	23 (24.2%)	16 (16.7%)	39 (20.4%)
Missing	1	0	1
Performance status (ECOG) [n (%)]			
Number	95	94	189
0	93 (97.9%)	91 (96.8%)	184 (97.4%)
1, 2	2 (2.1%)	3 (3.2%)	5 (2.6%)
Missing	1	2	3
Years from diagnosis to study entry			
Number	88	88	176
Mean (SD)	5.4 (3.4)	5.9 (3.9)	5.7 (3.7)
Missing	8	8	16

## Data Availability

All data necessary to evaluate and draw conclusions presented in this study are made readily available in the publication or as Supplementary Materials.

## Supplementary Materials

The following supporting information can be downloaded at: https://www.mdpi.com/article/10.3390/vaccines13040390/s1, Table S1: Inclusion and exclusion criteria of the phase II double-blind randomized trial; Table S2: List of antibodies used for flow cytometry analysis; Table S3: Active treatment group (n = 28). Ex vivo IFN-γ ELISpot RV001-specific spot counts and results of positivity test per visit and patient; Table S4: Active treatment group (n = 28). IVASS IFN-γ ELISpot RV001-specific spot counts and results of positivity test per visit and patient; Table S5: Placebo treatment group (n = 10). Ex vivo IFN-γ ELISpot RV001-specific spot counts and results of positivity test per visit and patient; Table S6: Placebo treatment group (n = 10). IVASS IFN-γ ELISpot RV001-specific spot counts and results of positivity test per visit and patient; Table S7: FM3, healthy donor 2 and patient 101-003 HLA-class II haplotypes; Figure S1: Vaccination schedule indicating timepoints for visits (V), vaccinations (#) and blood samples; Figure S2: (A) Schematic protocol overview for establishing and testing RV001-specific T-cell cultures. DCs, dendritic cells; ICS, intracellular cytokine staining; PBMCs, peripheral blood mononuclear cells. (B) Gating strategy for the ICS analysis to identify RV001-specific T cells; Figure S3: Gating strategy for the TCRBV flow cytometry panel; Figure S4: Screening of RV001-specific CD4^+^ clones of HD2; Figure S5: Normalized cell index for FM3 and peptide-pulsed FM3 (pFM3) alone and in co-culture with clone 1B5 at effector to target ratio 10:1, 3:1, and 1:1; Figure S6: Normalized cell index for FM3, FM3 + anti HLA-II antibody, and peptide-pulsed FM3 (pFM3) alone and in co-culture with the T cell line derived from patient 101-003 at effector to target ratio 10:1, 3:1, and 1:1; Figure S7: (A,B) Uncropped full scans of western blots from the corresponding cropped western blots shown within the Figure 5; Figure S8: Uncropped full scans of gels from the corresponding gels shown within the Figure 7.

## Appendix A

### List of RhoVac II Principal Investigators

Denmark:

Niels-Christian Langkilde, Aalborg University Hospital

Mads Hvid-Poulsen, Odense University Hospital

Michael Borre, Aarhus University Hospital

Lisa Andersen, Herlev & Gentofte Hospital

Jørgen Bjerggaard Jensen, Hospital Unit West, Holstebro

Finland:

Teuvo Tammela, Tampere University Hospital

Antti Rannikko, Helsinki University Hospital

Peter Broström, Turku University Hospital

Timo Martilla, Seinajoki Central Hospital

Hanna Ronkainen, Oulu University Hospital

Sweden:

Enrique Castellanos, Karolinska University Hospital, Stockholm

Anders Bjartell, Skåne University Hospital, Malmø

Johan Stranne, Sahlgrenska University Hospital, Göteborg

Börje Ljungberg, Umeå University Hospital

Janusz Frey, University Hospital Örebro

Belgium:

Nicolaas Lumen, Gent University Hospital

David Waltregny, CHU Liège Sart-Tilman, Liege

Thierry Roumeguere, Hospital Erasme, Bruxelles.

Germany:

Tilman Todenhöfer, Studienpraxis Urologie, Nürtigen

Arnulf Stenzl, Universitätsklinikum, Tübingen

Wolfgang Warnack, Urologische Arztpraxis, Hagenow

Christian Thomas, Klinik und Poliklinik für Urologie, Universitätsklinikum „Carl Gustav Carus“ der Technischen Universität Dresden

Eva Hellmis, Urologicum Duisburg, Duisberg

Miroslav Markov, Urologische Praxis, Halle/Saale, Germany

United Kingdom:

Simon Crabb, University Hospital Southampton NHS Foundation Trust

Zafar Malik, The Clatterbridge Cancer Centre NHS Foundation Trust, Bebington

Sarah Needleman, Royal Free London NHS Foundation Trust

Eliot Chadwick, Nottingham University Hospitals NHS Trust

United States of America:

Richard Levin, Chesapeake Urology Research Associates, Annapolis, Maryland

Nicholas Vogelzang, US Oncology Research Comprehensive Cancer Centers of Nevada, Las Vegas

Neal Shore, Carolina Urologic Research Center, Myrtle Beach, South Carolina

Luke Nordquist, GU Research Network/Urology Cancer Center, Omaha, Nebraska

Ashutosh Tewari, Icahn School of Medicine at Mount Sinai, New York

James Mark Zachary, Tampa Bay Medical Research, Inc. Clearwater, Florida

Naveen Kella, The Urology Place, San Antonio, Texas
