# Supplementary material for: Vaccination Against RhoC in Prostate Cancer Patients Induces Potent and Long-Lasting CD4+ T Cell Responses with Cytolytic Potential in the Absence of Clinical Efficacy: A Randomized Phase II Trial
Source: Vaccines (Basel). 2025 Apr 5;13(4):390. doi: 10.3390/vaccines13040390 (PMC12031432; doi:10.3390/vaccines13040390)
Supplement: Supplementary file 1 [file vaccines-13-00390-s001.zip › vaccines-3464587-supplementary.pdf]

Table S1. Inclusion and exclusion criteria of the phase II double-blind randomized trial.

Inclusion criteria	Exclusion criteria
Men aged 18 and above with an earlier histologic diagnosis of prostatic adenocarcinoma.	Patients who are receiving androgen-deprivation therapy (ADT) or considered a candidate for immediate ADT or are candidate to any local therapy according to applicable clinical guidelines or judged by the investigator.<br>Patients who have received prior ADT are not eligible with the exception of those that received ADT ≤36 months in duration and ≥9 months before randomization and administered only in the neoadjuvant/adjuvant setting
Able to understand the study procedures and willing to provide informed consent.	Patient is planned for salvage therapy.
Able and willing to comply with study requirements and complete all visits.	Castrate level of serum testosterone <50 ng/dL at screening
Biochemical recurrence (BCR) in compliance with the following 3 conditions:<br>• after having finished last definitive treatment (including those who have received radical prostatectomy (RP)/radiation therapy (RT) followed by any modality of salvage therapy)<br>• no distant metastasis by standard CT imaging with bone scintigraphy or a normal PET-CT<br>• no locoregional recurrence (including lymph nodes). Locoregional recurrence will be assessed by multi-parametric MRI in all patients treated with curative RT and should be confirmed with image guided prostatic gland biopsy in case of suspicious lesions. Any prostatic biopsy performed should be negative.	PSA > 10 ng/mL
Prior definitive treatment with RP or RT. In case the patient was subjected to a RP, all the following will apply:<br>• PSA ≥0.2 ng/mL,<br>• PSA doubling time (PSADT)[a] >3 months and <12 months<br>• History of Gleason score of 7 (4 + 3) or higher<br>Conversely, if patient was treated with definitive RT and not prior RP, all the following apply:<br>• PSA > nadir + 2 ng/mL<br>• PSADT >3 months and <12 months<br>• History of Gleason score of 7 (4 + 3) or higher	Small-cell, signet cell and neuroendocrine variants of adenocarcinomas.
Eastern Cooperative Oncology Group (ECOG) performance status ≤2.	An active malignancy likely to interfere with protocol treatment or follow-up.

<table>
<tr><td>Laboratory values obtained ≤30 d prior to first vaccination
Hemoglobin ≥5.6 mmol/L (0.72 g/L)
Absolute granulocyte count ≥1.5 x 109 /L.
Platelets ≥100 x 109 /L.
Total bilirubin ≤1.5 x upper limit of normal (ULN)
Creatinine ≤1.5 x ULN.
Alanine aminotransferase (ALT), aspartate aminotransferase (AST) and alkaline phosphatase (ALP) ≤2.5 x ULN.</td><td>Patients who have undergone major surgery or have had major bleeding within the last month prior to the first vaccination.</td></tr>
<tr><td></td><td>Prior treatment with any therapeutic cancer vaccine(s).</td></tr>
<tr><td></td><td>Participants with a condition requiring systemic treatment with either corticosteroids (>10 mg daily prednisone equivalent) or other immunosuppressive medications within 14 d of randomization. Inhaled or topical steroids, and adrenal replacement steroid doses >10 mg daily prednisone equivalent, are permitted in the absence of active autoimmune disease.</td></tr>
<tr><td></td><td>History of alcohol or substance abuse within the last 5 years.</td></tr>
<tr><td></td><td>Patients receiving any investigational drug(s) or treatment within 30 d prior to inclusion in this trial.</td></tr>
<tr><td></td><td>History of significant autoimmune disease such as inflammatory bowel disease, systemic lupus erythematosus, ankylosing spondylitis, scleroderma, multiple sclerosis (MS).</td></tr>
<tr><td></td><td>Severe medical conditions, such as but not limited to severe asthma/chronic obstructive pulmonary disease (COPD), New York Heart Association (NYHA) grading 3 or above, poorly regulated insulin dependent diabetes, any significant organ damage as judged by the investigator.</td></tr>
<tr><td></td><td>Other medications, conditions or laboratory results that in the investigator's opinion would contraindicate study participation for safety reasons or interfere with the interpretation of study results.</td></tr>
<tr><td></td><td>History of known allergy/hypersensitivity to any component of the study drug (such as Montanide ISA 51), or intolerance to subcutaneous (SC) injection.</td></tr>
<tr><td></td><td>Patients with a prior solid organ/stem cell transplantation.</td></tr>
<tr><td></td><td>Patients with known acquired immunodeficiency disorder (AIDS) or any inherited immunodeficiency disorder</td></tr>
</table>

Table S2. List of antibodies used for flow cytometry analysis.

NAME	SUPPLIER	REACTIVITY	ISOTYPE	CLONE	CAT. NUMBER	DILUTION
CD107a-BV421	BD	Human	IgG1 k	H4A3	566261	1:666
CD3-AF700	BioLegend	Human	IgG1 k	UCHT1	317340	1:40
CD4-BV711	BioLegend	Human	IgG2b k	OKT4	560768	1:10
CD8-BV605	BioLegend	Human	IgG1 k	SK1	344704	1:10
Granulysin-PE	Fisher Scientific	Human	IgG1 k	DH2	GRB05	1:20
Granzyme B-APC	BioLegend	Human	IgG1	GB11	311408	1:40
IFNγ-BV510	BioLegend	Human	IgG1 k	4S.B3	562438	1:25
LIVE/DEAD™ Fixable Near-IR Dead Cell Stain	Fisher Scientific	-	-	-	L23105	1:500

Table S3. Active treatment group (n = 28). Ex vivo IFN-γ ELISpot RV001-specific spot counts and results of positivity test per visit and patient.

**Patients**	**Pre-vaccination**		**Visit 8**		**Visit 13**	
	Spots	DFR(2x)	Spots	DFR(2x)	Spots	DFR(2x)
**104-001**	3	neg	6	neg		
**101-003**	1	neg	95	pos***		
**105-004**	0	neg	3	neg		
**103-001**	0	neg	171	pos***		
**104-002**	0	neg	27	pos***		
**501-005**	1	neg	25	pos***		
**105-014**	0	neg	33	pos***		
**101-019**	0	neg	4	neg		
**101-020**	0	neg	12	pos*		
**501-003**	0	neg	4	neg		
**103-004**	0	neg	14	pos***	8	neg
**105-003**	0	neg	8	neg	1	neg
**105-013**	1	neg	2	neg		
**103-013**	0	neg	8	neg		
**106-005**	0	neg	15	pos***	9	neg
**101-011**	0	neg	2	neg	8	neg
**101-005**	2	neg	0	neg	1	neg
**106-003**	1	neg	2	neg		
**105-006**	0	neg	5	neg		
**103-014**	0	neg	1	neg	0	neg
**106-001**	0	neg	0	neg		
**303-004**	0	neg	0	neg		
**105-009**	1	neg	0	neg		
**101-006**	1	neg	1	neg		
**105-015**	1	neg	0	neg		
**101-013**	0	neg	0	neg	8	neg
**103-006**	0	neg	1	neg		
**106-004**	0	neg	2	neg		

Patients are listed in the same order as in Figure 3. Specific counts (background subtracted) and results of the DFR(2x) permutation for response determination are shown with significant p values: * $p< 0.05$; ** $p < 0.01$; *** $p< 0.001$. Only responses with ≥10 spots/well were considered positive. For details, see Materials and Methods. Gray = not tested.

Table S4. Active treatment group (n = 28). IVASS IFN-γ ELISpot RV001-specific spot counts and results of positivity test per visit and patient.

Patients	Prevaccination		Visit 8		Visit 13	
	Spots	DFR(2x)	Spots	DFR(2x)	Spots	DFR(2x)
**104-001**	40	pos**	2,000#	pos***		
**101-003**	318	pos***	1,998#	pos***		
**105-004**	17	neg	1,995#	pos***		
**103-001**	47	neg	1,997#	pos***		
**104-002**	368	pos***	1,994#	pos***		
**501-005**	56	pos*	1,994#	pos***		
**105-014**	142	neg	1,990#	pos***		
**101-019**	196	neg	1,951#	pos***		
**101-020**	135	neg	1,766#	pos***		
**501-003**	134	neg	1,562	pos***		
**103-004**	309	neg	1,415	pos***	1,994#	pos***
**105-003**	15	neg	1,387	pos***	1,082	pos***
**105-013**	18	neg	1,329	pos***		
**103-013**	58	neg	1,870#	pos***		
**106-005**	61	neg	1,298	pos***	1,269	pos***
**101-011**	33	neg	960	pos***	172	pos***
**101-005**	16	neg	765	pos***	1,949#	pos***
**106-003**	1	neg	568	pos***		
**105-006**	161	neg	546	pos***		
**103-014**	0	neg	475	pos***	357	pos***
**106-001**	2	neg	376	pos***		
**303-004**	27	neg	302	pos***		
**105-009**	10	neg	294	pos***		
**101-006**	62	neg	278	pos***		
**105-015**	19	neg	162	pos***		
**101-013**	106	neg	31	neg	94	neg
**103-006**	42	neg	9	neg		
**106-004**	24	neg	0	neg	5	neg

Patients are listed in the same order as in Figure 3. Specific counts (background subtracted) and results of the DFR(2x) permutation for response determination are shown with significant p values: * p< 0.05; ** p < 0.01; *** p< 0.001. For details, see materials and Methods. #TNTC wells set to 2,000. Gray = not tested.

Table S5. Placebo treatment group (n = 10). Ex vivo IFN-γ ELISpot RV001-specific spot counts and results of positivity test per visit and patient.

Patients	Pre-vaccination		Visit 8		Visit 13	
	Spots	DFR(2x)	Spots	DFR(2x)	Spots	DFR(2x)
**501-006**	4	neg	6	neg		
**103-007**	0	neg	1	neg		
**105-002**	0	neg	0	neg	0	neg
**104-011**	0	neg	0	neg	1	neg
**101-004**	0	neg	0	neg		
**103-011**	1	neg	1	neg	0	neg
**303-003**			0	neg		
**101-016**	0	neg	1	neg	0	neg
**103-005**	0	neg	0	neg	0	neg
**101-009**	0	neg	2	neg	0	neg

Patients are listed in the same order as in Figure 3. Specific counts (background subtracted) and results of the DFR(2x) permutation for response determination are shown. Only responses with ≥10 spots/well were considered positive. For details, see Materials and Methods. Gray = not tested.

Table S6. Placebo treatment group (n = 10). IVASS IFN-γ ELISpot RV001-specific spot counts and results of positivity test per visit and patient.

Patients	Pre-vaccination		Visit 8		Visit 13	
	Spots	DFR(2x)	Spots	DFR(2x)	Spots	DFR(2x)
**501-006$**	750	pos***	1,238	pos***		
**103-007$**	536	pos**	150	pos***		
**105-002**	4	neg	96	pos***	258	neg
**104-011**	128	neg	74	neg	207	neg
**101-004**	43	neg	63	neg		
**103-011**	6	neg	0	neg	0	neg
**303-003**	12	neg	36	neg	155	pos***
**101-016**	68	neg	34	neg	91	neg
**103-005**	14	neg	1	neg	37	neg
**101-009**	54	neg	0	neg	2	neg

Patients are listed in the same order as in Figure 3. Specific counts (background subtracted) and results of the DFR(2x) permutation for response determination are shown with significant p values: * p< 0.05; ** p < 0.01; *** p< 0.001. For details, see Materials and Methods. $: T cell reactivity not boosted. Gray = not tested

Table S7. FM3, healthy donor 2 and patient 101-003 HLA-class II haplotypes.

	DRB1 (1)	DRB1 (2)	DQB1 (1)	DQB1 (2)	DPB1 (1)	DPB1 (2)
**FM3**	15:01			06:02	04:01	
**HD2**	11:01	13:02	03:01	06:04	04:02:01	03:01:01
**Pat-101-003**	07:01:01G	13:01	03:03	06:03	04:01	03:01

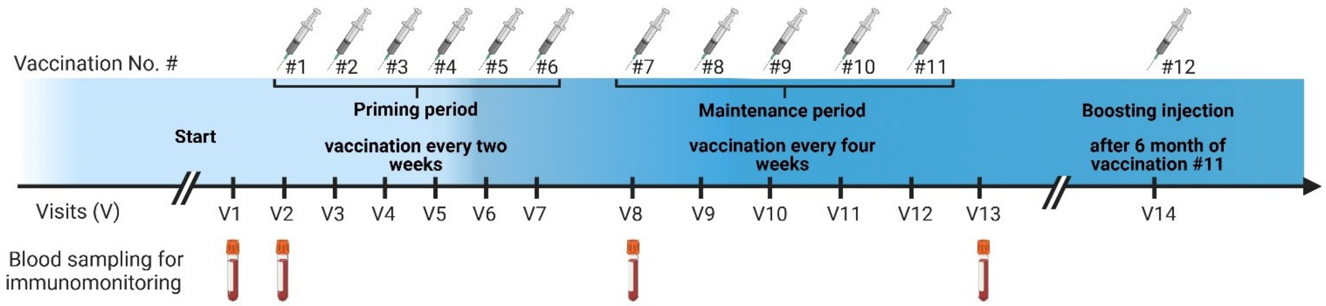

Figure S1. Vaccination schedule indicating timepoints for visits (V), vaccinations (#) and blood samples. Blood for immunomonitoring was obtained at visits V1 or V2 (pre-vaccination), visit V8 (after the 6 priming vaccinations) and at visit V13 (after the 11 priming and maintenance vaccinations). Created with Biorender.

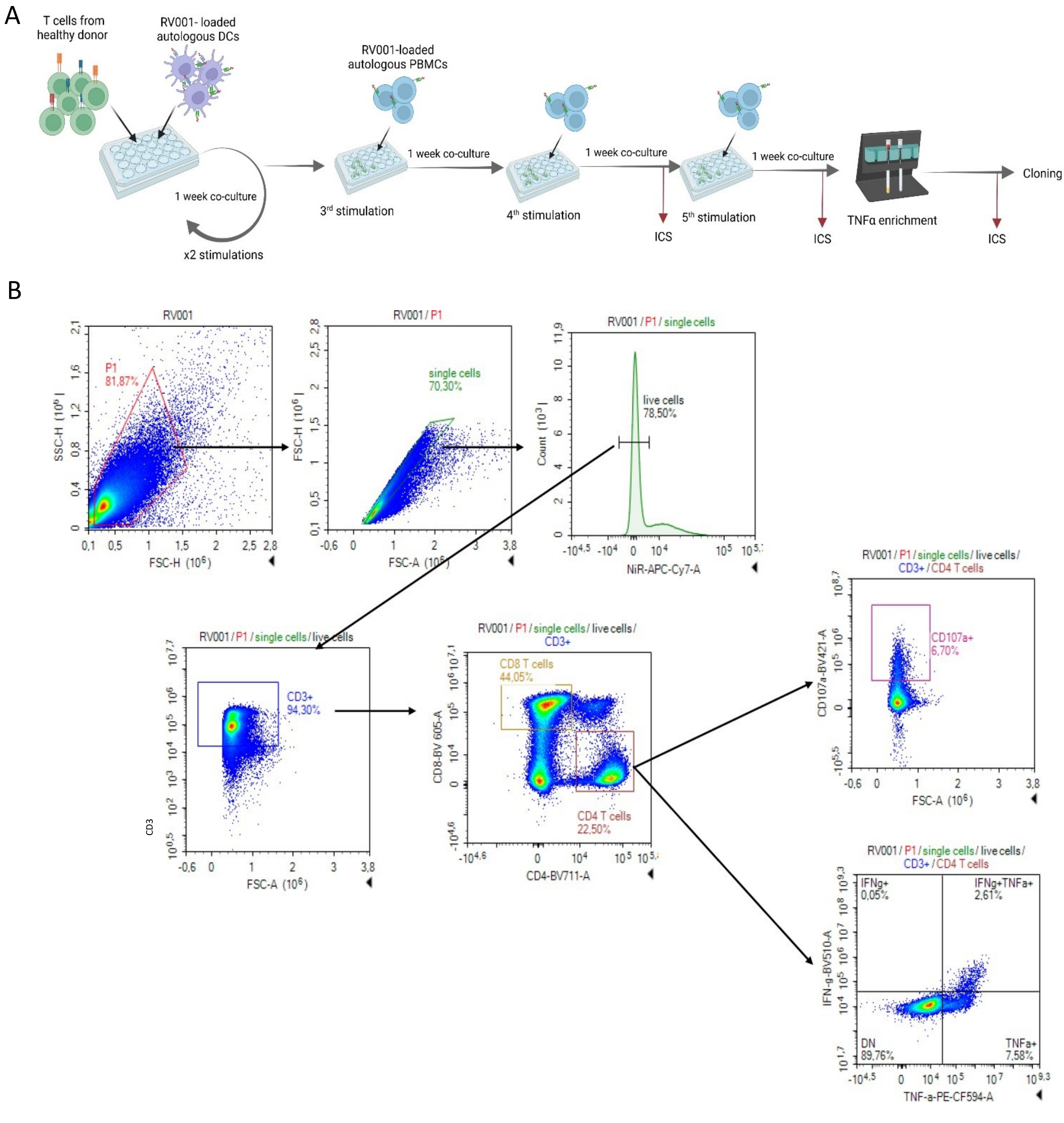

Figure S2. (A) Schematic protocol overview for establishing and testing RV001-specific T-cell cultures. DCs, dendritic cells; ICS, intracellular cytokine staining; PBMCs, peripheral blood mononuclear cells. (B) Gating strategy for the ICS analysis to identify RV001-specific T cells. Upper row, left to right: Cell population selection based on size and granularity using side scatter height (SSC-H) and forward scatter height (FSC-H), doublet exclusion using forward scatter area (FSC-A) versus FSC-H, live cell gating using Near-Infrared (NIR) Live/Dead viability dye detected in the APC-Cy7 channel (NIR-APC-Cy7-A), T lymphocyte selection based on FSC-A and CD3 expression (CD3-AlexaFluor700-A). CD4 and CD8 populations were distinguished using CD4 (CD4-BV711-A) and CD8 (CD8-BV605-A). CD4+ T cells were analyzed for CD107a (CD107a-BV421-A) to assess degranulation and for TNF-α (TNF-a -PE-CF594-A) and IFN-γ (IFN-g-BV510-A) to evaluate cytokine production.

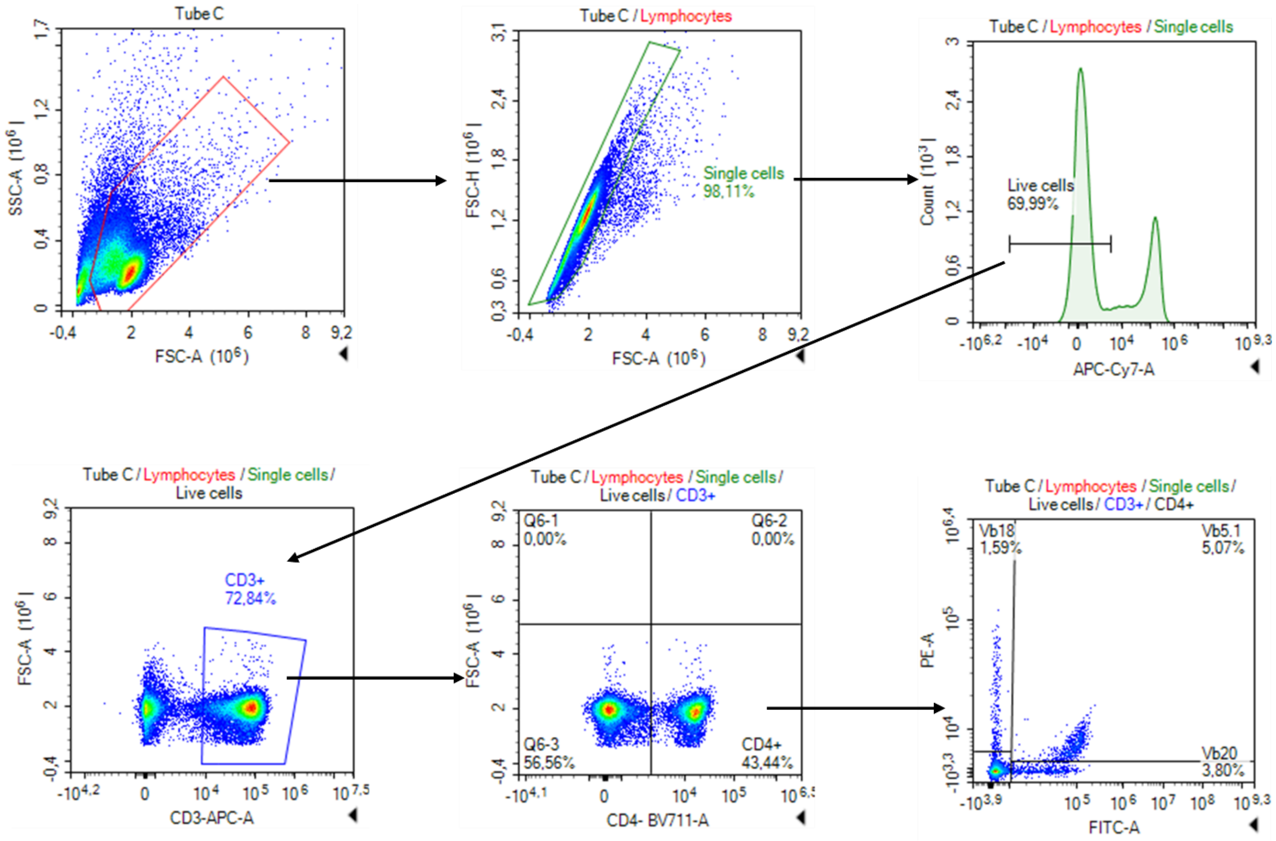

Figure S3. Gating strategy for the TCRBV flow cytometry panel. Upper row, left to right: Cell population selection based on size and granularity using side scatter area (SSC-A) and forward scatter area (FSC-A), doublet exclusion using forward scatter area (FSC-A) versus forward scatter height (FSC-H), live cell gating using Near-Infrared (NIR) Live/Dead viability dye detected in the APC-Cy7 channel (APC-Cy7-A), T lymphocyte selection based on CD3 expression (CD3-APC-A), $CD4^+$ T cell selection based on CD4 expression (CD4-BV711-A). $CD4^+$ T cells analyzed for TCRBV chain expression, with BV18 (PE-A), BV20 (FITC-A), and BV5.1 (PE/FITC double positives).

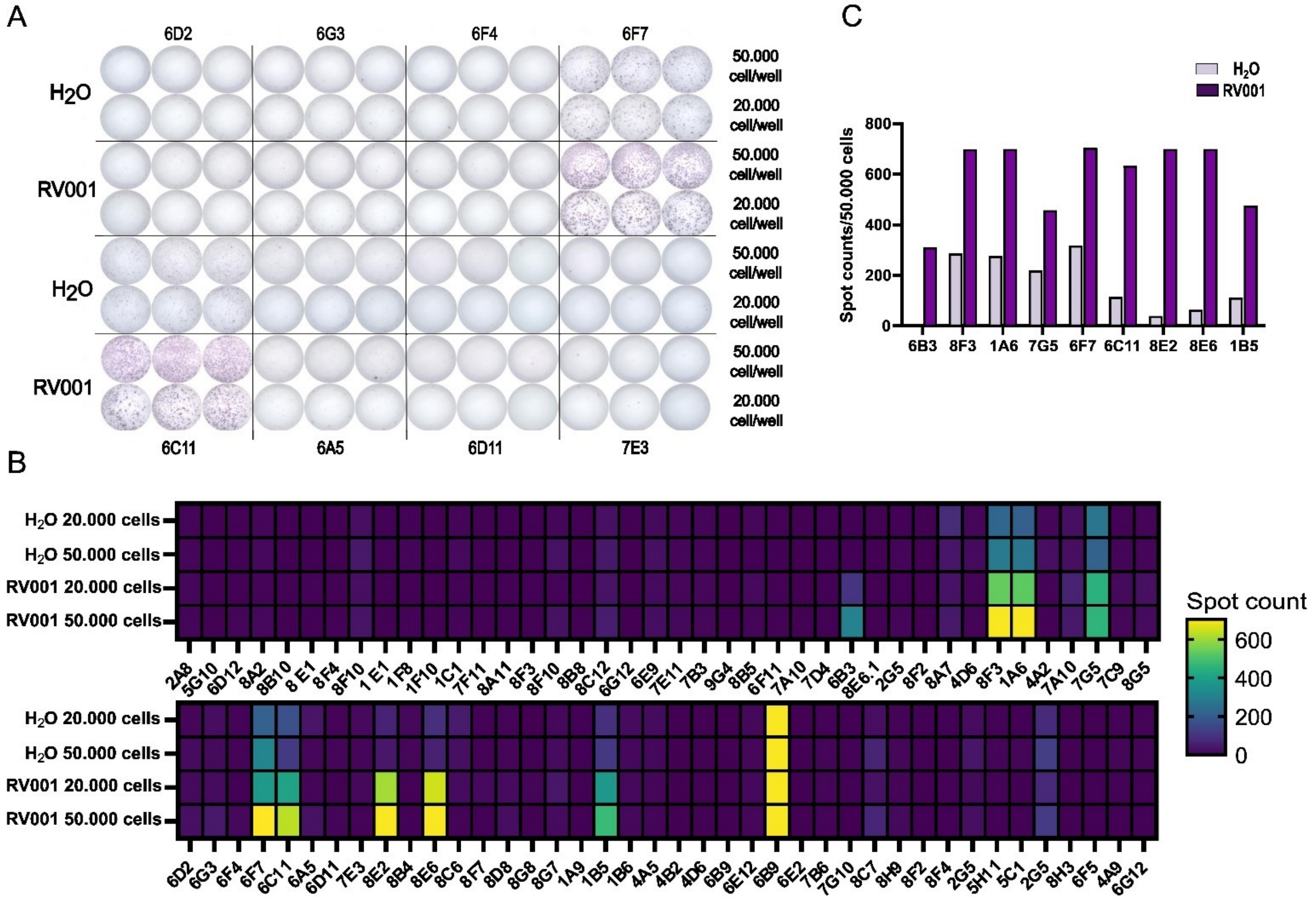

Figure S4. Screening of RV001-specific CD4+ clones of HD2. Growing clones were screened and selected for peptide-reactivity with an IFNγ ELISPOT assay using the DFR method. 20,000 and 50,000 cells of each clone were cultured for 24 h in the presence of RV001 (1μM) diluted in distilled H_2O, or the same amount of distilled H_2O as a negative control. A) Representative example of an ELISpot plate containing 8 clones. B) Heatmap depicting mean spot counts of all clones for the peptide and the negative control conditions. C) Histogram of IFNγ responses in the selected clones showing spot counts/50,000 cells. Bars represent mean spot numbers for RV001 and negative control wells. All tests were performed in triplicates.

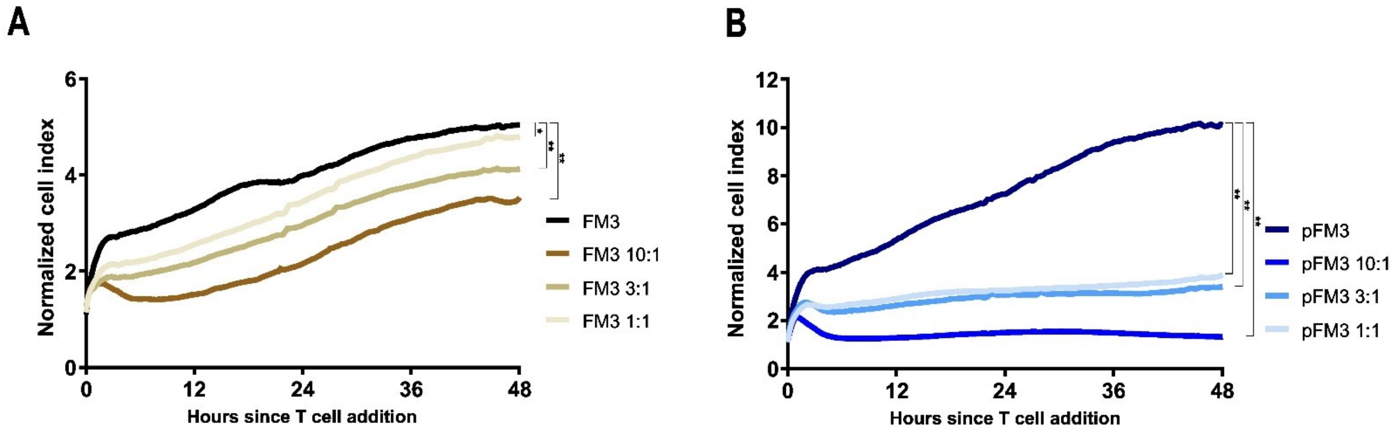

Figure S5. Normalized cell index for FM3 and peptide-pulsed FM3 (pFM3) alone and in co-culture with clone 1B5 at effector to target ratio 10:1, 3:1, and 1:1. * indicates $p < 0.05$ and ** indicates $p < 0.01$.

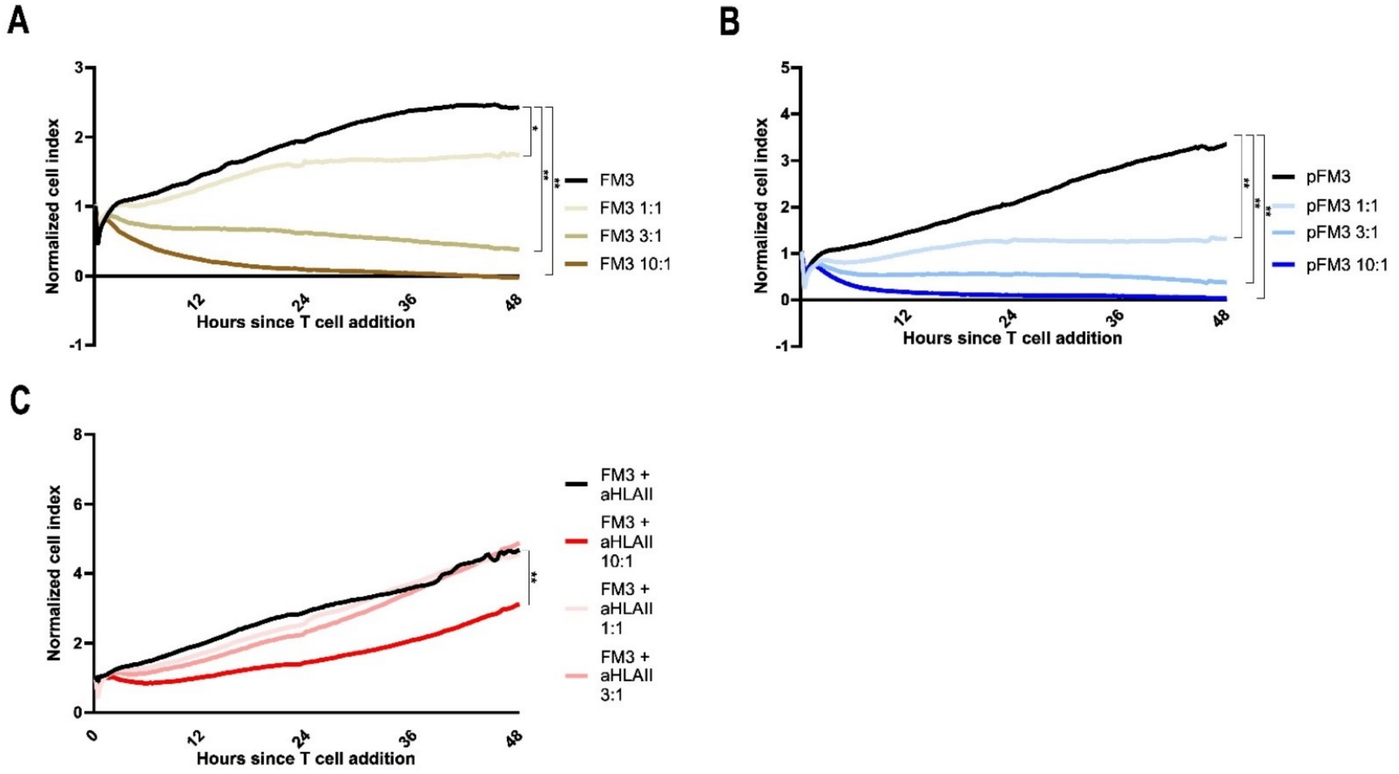

Figure S6. Normalized cell index for FM3, FM3 + anti HLA-II antibody, and peptide-pulsed FM3 (pFM3) alone and in co-culture with the T cell line derived from patient 101-003 at effector to target ratio 10:1, 3:1, and 1:1. * indicates $p < 0.05$ and ** indicates $p < 0.01$.

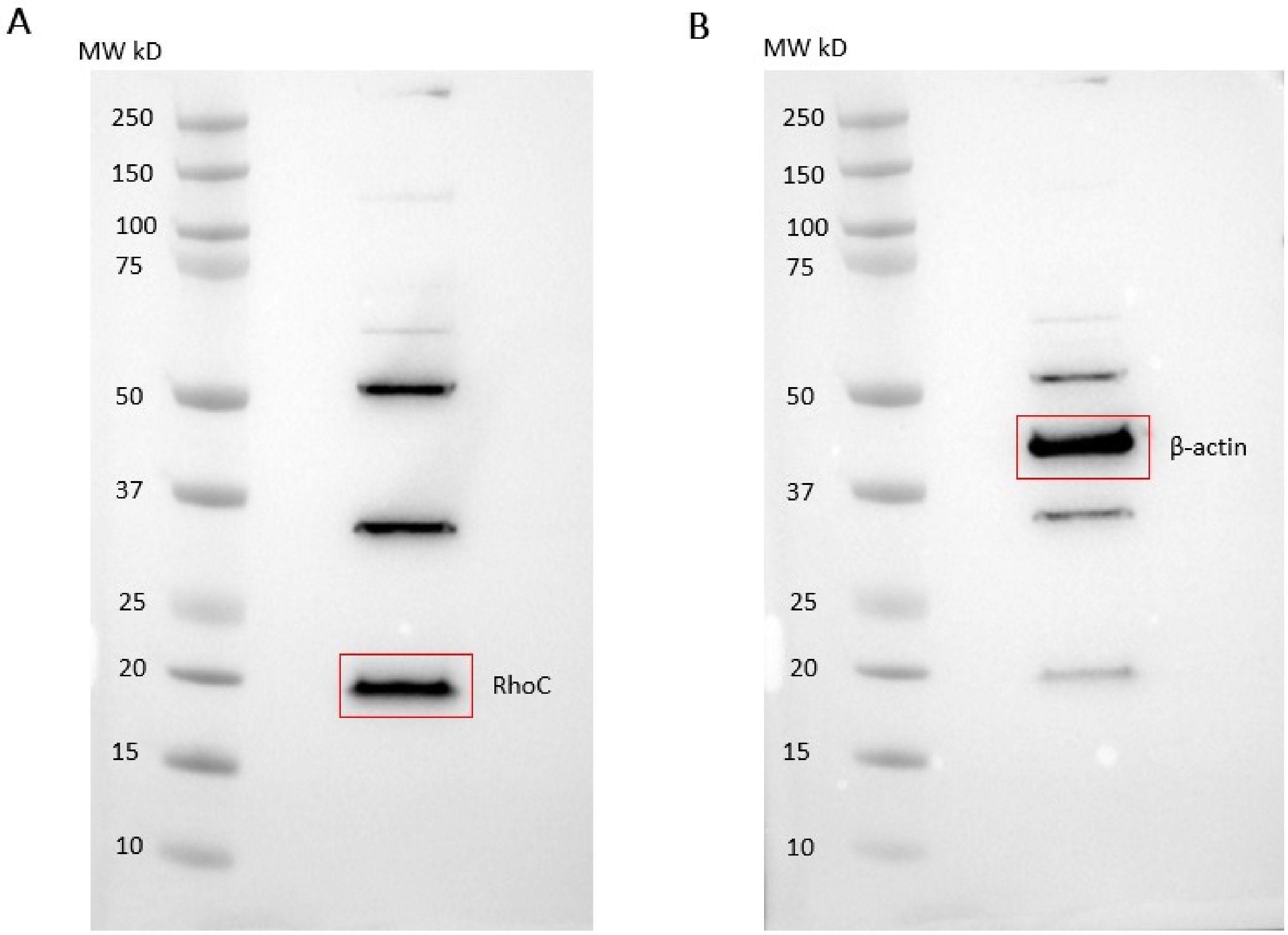

Figure S7. (A,B) Uncropped full scans of western blots from the corresponding cropped western blots shown within the Figure 5. Molecular weight markers are indicated. Densitometry reading was 1,510,000 and 1,840,000 for RhoC and β-actin respectively (calculated with Image Studio Lite).

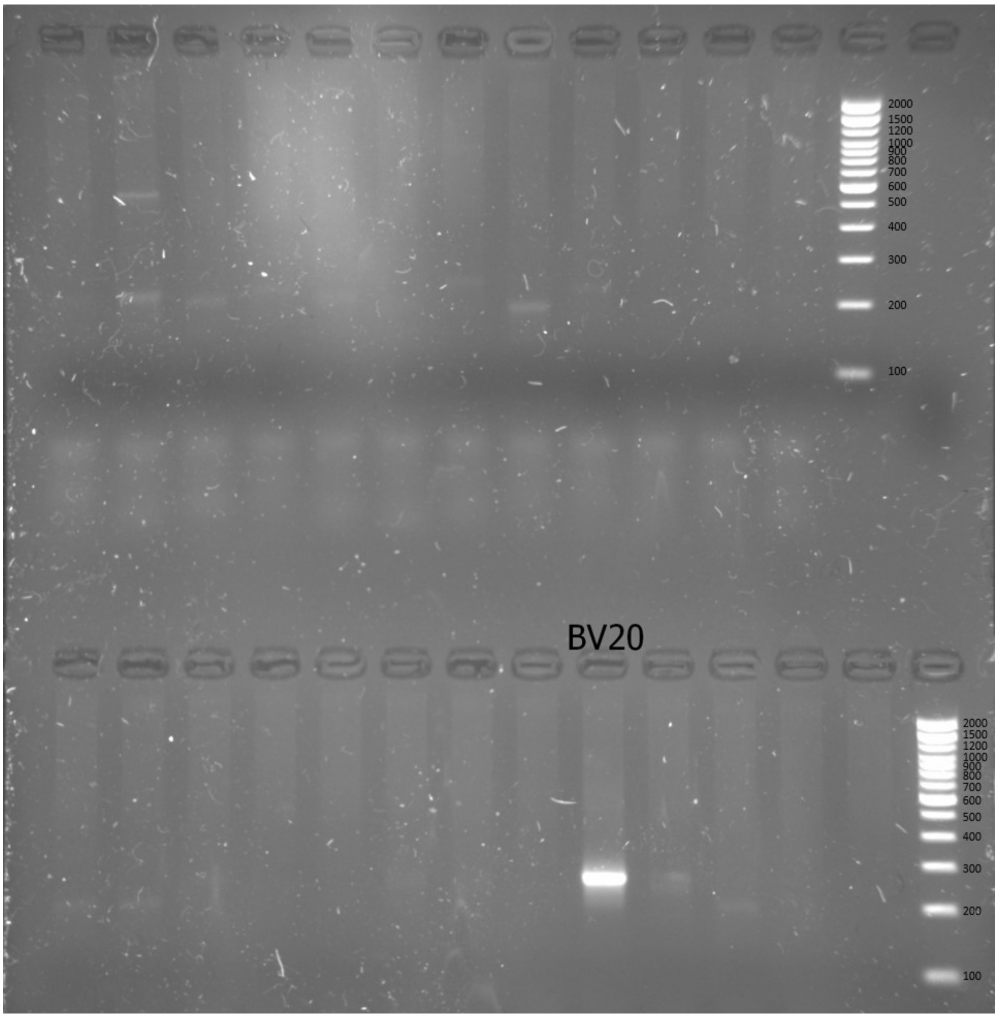

Figure S8. Uncropped full scans of gels from the corresponding gels shown within the Figure 7.
